# Effect of Pregnenolone vs Placebo on Self-reported Chronic Low Back Pain Among US Military Veterans: A Randomized Clinical Trial

**DOI:** 10.1001/jamanetworkopen.2020.0287

**Published:** 2020-03-02

**Authors:** Jennifer C. Naylor, Jason D. Kilts, Lawrence J. Shampine, Gillian J. Parke, H. Ryan Wagner, Steven T. Szabo, Karen D. Smith, Trina B. Allen, Emily G. Telford-Marx, Charlotte E. Dunn, Brian T. Cuffe, Susan H. O’Loughlin, Christine E. Marx

**Affiliations:** 1 Durham VA Health Care System, Durham, North Carolina; 2 Department of Psychiatry and Behavioral Sciences, Duke University Medical Center, Durham, North Carolina; 3 VA Mid-Atlantic Mental Illness, Research, Education and Clinical Center, Durham, North Carolina

## Abstract

**Importance:**

In response to the national opioid public health crisis, there is an urgent need to develop nonopioid solutions for effective pain management. Neurosteroids are endogenous molecules with pleotropic actions that show promise for safe and effective treatment of chronic low back pain.

**Objective:**

To determine whether adjunctive pregnenolone has therapeutic utility for the treatment of chronic low back pain in Iraq- and Afghanistan-era US military veterans.

**Design, Setting, and Participants:**

Randomized, double-blind, placebo-controlled clinical trial that enrolled for 42 months, from September 2013 to April 2017. Participants were Iraq- and Afghanistan-era veterans aged 18 to 65 years with chronic low back pain who received treatment in the Durham VA Health Care System in Durham, North Carolina, over 6 weeks. Data analysis began in 2018 and was finalized in March, 2019.

**Interventions:**

Following a 1-week placebo lead-in, participants were randomized to pregnenolone or placebo for 4 weeks. Pregnenolone and placebo were administered at fixed, escalating doses of 100 mg for 1 week, 300 mg for 1 week, and 500 mg for 2 weeks.

**Main Outcomes and Measures:**

The primary outcome measure was the change in mean pain intensity ratings from a daily pain diary (numerical rating scale, 0-10) between visit 3 (baseline) and visit 6. Secondary outcomes included pain interference scores (Brief Pain Inventory, Short Form). Preintervention and postintervention neurosteroid levels were quantified by gas chromatography with tandem mass spectrometry. Hypotheses tested were formulated prior to data collection.

**Results:**

A total of 94 participants (84 [89.4%] male; mean [SD] age, 37.5 [9.8] years; 53 [56.4%] of self-reported Caucasian race and 31 [33.0%] of self-reported African American race) were included. Forty-eight participants were randomized to pregnenolone and 52 to placebo, of whom 45 and 49, respectively, were included in baseline demographic characteristics secondary to noncompliance with medications as per protocol. Veterans randomized to pregnenolone reported significant reductions in low back pain relative to those randomized to placebo. Baseline unadjusted mean (SE) pain diary ratings were 4.83 (0.23) and 5.24 (0.22) for the placebo- and pregnenolone-treated groups, respectively (baseline unadjusted mean [SE] ratings for pain recall were 4.78 [0.24] and 5.15 [0.23], respectively). Unadjusted mean (SE) ratings following treatment (visit 6) were 4.74 (0.26) in the placebo group and 4.19 (0.30) in the pregnenolone-treated group. Unadjusted mean (SE) ratings for pain recall following treatment were 4.86 (0.27) for placebo and 4.18 (0.29) for pregnenolone. Least-square mean (LSM) analysis showed that pain scores significantly improved in the pregnenolone-treated group compared with placebo (LSM [SE] change in pain diary rating, −0.56 [0.25]; *P* = .02; LSM [SE] change in pain recall, −0.70 [0.27]; *P* = .01). Pain interference scores for work (LSM [SE] change, 0.71 [0.12]; *P* = .04) and activity (LSM [SE] change, 0.71 [0.11]; *P* = .03) were also improved in veterans randomized to pregnenolone compared with placebo. Pregnenolone was well tolerated.

**Conclusions and Relevance:**

Participants receiving pregnenolone reported a clinically meaningful reduction in low back pain and 2 pain interference domains compared with those receiving placebo. Pregnenolone may represent a novel, safe, and potentially efficacious treatment for the alleviation of chronic low back pain in Iraq- and Afghanistan-era veterans.

**Trial Registration:**

ClinicalTrials.gov Identifier: NCT01898013

## Key Points

**Question:**

Does adjunctive treatment with pregnenolone improve chronic low back pain in Iraq- and Afghanistan-era US military veterans?

**Findings:**

The use of pregnenolone in this randomized, double-blind, placebo-controlled clinical trial in 94 veterans with chronic low back pain resulted in a significant reduction in pain intensity ratings after 4 weeks of treatment.

**Meaning:**

These findings suggest that pregnenolone may be a safe and effective adjunctive treatment for veterans with chronic low back pain.

## Teaser

This randomized clinical trial investigates the use of pregnenolone vs placebo for low back pain among Iraq- and Afghanistan-era US military veterans.

## Introduction

The development of nonopioid pharmacological interventions that maximize analgesia and function while minimizing adverse outcomes has become a national imperative, as overreliance on prescription opioid medications has played a substantial role in the recent surge of opioid-related addiction and overdose deaths. New treatment modalities for pain are thus urgently needed, and neurosteroids hold promise in this regard as safe^1,2,3,4,5^ and effective approaches for the treatment of chronic pain.

Neurosteroids are endogenous molecules enriched in the brain that exhibit pleiotropic actions in the central nervous system. Allopregnanolone, a positive GABA_A_ (γ-aminobutyric acid) receptor modulator^6,7^ and downstream metabolite of pregnenolone, has known analgesic,^8,9,10,11,12,13,14^ neuroprotective,^15,16,17,18,19,20,21^ neurotrophic,^18^ and anti-inflammatory properties.^22,23,24^ Extensive data from preclinical models demonstrate that neurosteroids exhibit pronounced analgesic actions, and data demonstrate that neurosteroids are decreased in the setting of pain symptoms in clinical populations.^25,26^ Thus, restoration of low or depleted endogenous levels of neurosteroids such as allopregnanolone may have therapeutic utility for chronic low back pain. We therefore hypothesized that adjunctive pregnenolone would reduce pain ratings in Iraq- and Afghanistan-era US military veterans with chronic low back pain.

## Methods

### Study Design

This randomized, double-blind, placebo-controlled clinical trial sought to determine whether adjunctive pregnenolone reduces chronic low back pain in Iraq- and Afghanistan-era veterans. The study took place in Durham, North Carolina, and its total duration was 6 weeks. Reporting of the trial follows the Consolidated Standards of Reporting Trials (CONSORT) reporting guideline. The Durham Veterans Affairs (VA) Health Care System institutional review board approved the trial protocol (available in Supplement 1). Written informed consent and Health Insurance Portability and Accountability Act authorization were obtained from all participants.

### Data Collection

Eligible participants completed daily pain diaries for 1 week (and throughout study participation), then completed a 1-week placebo-only lead-in period. At the third study visit, participants were randomized to receive pregnenolone or matching placebo for 4 weeks. Participants returned pain diaries and completed interviews, self-reported assessments, and standard laboratory studies (nonfasting complete blood count, basic metabolic panel [CHEM-7], and gastrointestinal panel) at each interview. Electrocardiograms were completed before and after treatment to ensure that there were no cardiac concerns for enrollment or as a result of study participation. Participants were contacted for follow-up by telephone 1 and 2 weeks after study completion.

### Participants and Disease Diagnostic Criteria

Eligible participants were Iraq- and Afghanistan-era veterans (age 18-65 years) with chronic low back pain, defined as pain most days for the preceding 6 months or longer, and a weekly mean intensity score of 4 or greater at baseline. Pain was required to be either restricted to thoracic vertebrae 6 or below or associated with radiation to the proximal portion of the lower limb only. Study participants could not have (1) neurological radicular signs, (2) presumptive compression of a spinal nerve root on a simple radiogram, or (3) compression of a spinal nerve root confirmed by specific imaging or other diagnostic techniques. The aforementioned diagnoses were confirmed by the study physician and based on history and physical and neurological examination. Additional exclusion criteria were spinal fracture, spondylolisthesis grade 3 or 4, tumor, and abscess or acute pathology in the low back or abdominal regional, as confirmed by historical record of imaging studies.

Other eligibility requirements included stable medication regimen and therapeutic interventions for 4 weeks prior to study entry and throughout study duration. Participants were required to ambulate without assistive devices and could not have received epidural steroids, facet blocks, nerve blocks, or other invasive procedures aimed to reduce low back pain within 3 months of study start. Participants were excluded for unstable medical or neurological illness; clinically meaningful suicidal and/or homicidal ideation; diagnosis of bipolar disorder, schizophrenia, or other psychotic disorders; cognitive disorder due to a general medical condition; or daily use of long- or short-acting opioid medications. History of mild traumatic brain injury was permitted. Treatment compliance was defined as taking between 80% and 120% of the study drug prescribed for that interval.

### Randomization and Interventions

**Figure 1.**
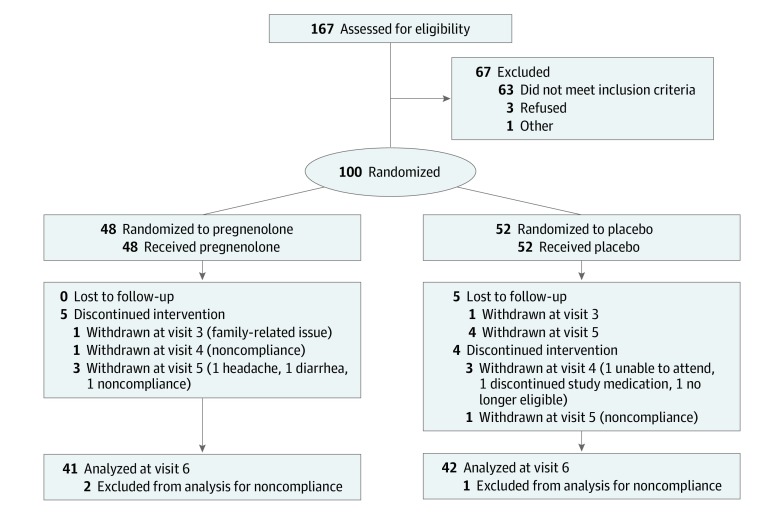
CONSORT Study Flow Diagram Treatment compliance was defined as taking between 80% and 120% of the study drug prescribed for that interval. Baseline demographic characteristics were thus calculated from 45 participants in the pregnenolone group and 49 participants in the placebo group (Table 1).

Eligible participants were randomized to treatment condition by the research pharmacist using the permuted block randomization strategy to assure equal balance between groups (Figure 1). Forty-eight participants were randomized to pregnenolone and 52 to placebo, of whom 45 and 49, respectively, were included in the baseline demographic characteristics (3 participants were withdrawn from each group secondary to noncompliance with medications as per protocol) (Table 1). Investigators and participants were blinded throughout study duration. Pregnenolone was prescribed at fixed escalating dosages up to 500 mg/d. Specifically, after a 1-week single-blinded placebo lead-in phase, participants randomized to pregnenolone received 100 mg (50 mg twice daily) for 1 week, 300 mg (150 mg twice daily) for 1 week, and 500 mg (250 mg twice daily) for 2 weeks. A 4-day taper was initiated at the final study visit. Pregnenolone and matching placebo were purchased from Green Mountain Pharmaceuticals.

**Table 1.** Participant Demographic Characteristics and Baseline Features

Characteristic	Complete Cohort (n = 94)	Placebo (n = 49)	Pregnenolone (n = 45)
Age, mean (SD), y	37.52 (9.84)	37.55 (10.25)	37.48 (9.47)
Sex, No. (%)			
Female	10 (10.6)	5 (50.0)	5 (50.0)
Male	84 (89.4)	44 (52.4)	40 (47.6)
Race/ethnicity, No. (%)			
Caucasian	53 (56.4)	29 (54.7)	24 (45.3)
African American	31 (33.0)	16 (51.6)	15 (48.4)
Other	10 (10.6)	4 (40.0)	6 (60.0)
Pretreatment pain intensity measures, mean (SD)			
Pain intensity (diary)	5.03 (1.55)	4.83 (1.60)	5.24 (1.48)
Pain recall	4.95 (1.59)	4.78 (1.66)	5.15 (1.51)
Pain intensity^a^	4.79 (1.54)	4.51 (1.39)	5.09 (1.66)
Pretreatment pain interference measures, mean (SD)			
Average interference^a^	2.89 (2.35)	2.49 (2.19)	3.33 (2.46)
Activity	3.35 (2.53)	2.78 (2.35)	3.98 (2.61)
Mood	2.79 (2.54)	2.47 (2.49)	3.13 (2.58)
Walking	2.80 (2.54)	2.29 (2.36)	3.36 (2.64)
Work	3.15 (2.63)	2.61 (2.40)	3.73 (2.77)
Relations	1.89 (2.35)	1.41 (2.02)	2.42 (2.59)
Sleep	3.52 (2.94)	3.43 (2.84)	3.62 (3.07)
Enjoyment	2.76 (2.70)	2.47 (2.51)	3.07 (2.90)

^a^ Measured using the Brief Pain Inventory.

### Outcome Measures

The primary efficacy outcome measure was the change in calculated mean of daily self-reported pain intensity scores on an 11-point numerical rating scale between visit 3 (baseline) and visit 6. The numerical rating scale was ordinal, ranging from 0 (no pain) to 10 (worst possible pain). During each study visit, participants were also asked to estimate their average low back pain score since their prior visit (pain recall). Secondary outcomes included pain interference scores from the well-validated Brief Pain Inventory, Short Form, a self-reported scale measuring pain severity and interference. Pain interference scores range from 0 (does not interfere) to 10 (completely interferes). There are 7 questions assessing interference of pain in the past 24 hours for general activity, mood, walking ability, work, relations with other people, sleep, and enjoyment of life. An overall pain interference score was calculated, as was each pain interference domain. Additional secondary outcome variables were assessed, including sleep (Athens Insomnia Scale), resilience (Connor-Davidson Resilience Scale), working memory (Digit Sequence), posttraumatic stress disorder (Davidson Trauma Scale), executive function (Tower of London), and physical function (RAND-36).

### Statistical Analysis

The current study was powered at 80% to detect a difference of 0.6 between groups with means of 6.0 at a significance level of .05. Data analysis began in 2018 and was completed in 2019. Nonparametric procedures were used to test differences related to randomization in baseline levels of demographic factors, psychological factors, and pain intensity and interference between treatment conditions. Categorical factors were tested using standard χ^2^ procedures, while ordinal and/or categorical measures were tested using nonparametric Wilcoxon Kruskal-Wallis procedures. Multivariable modeling of primary pain measures was conducted using longitudinal regression procedures. Thus, the pain diary rating was regressed on a model including the baseline (visit 3) pain rating, a dichotomous proxy denoting treatment status (placebo vs pregnenolone), visit, and an interaction term crossing treatment with visit. The test of the primary hypothesis was based on a contrast of the difference in estimated least-square means (LSMs) between the placebo and pregnenolone conditions at the final visit (visit 6). Subsequently, the latter test was repeated substituting the pain recall measure. Models were estimated using longitudinal generalized linear regression procedures (SAS version 9.4 [SAS Institute Inc]: SAS PROC GLIMMIX). Correlations between repeated measurements of the dependent variable were modeled using a first-order autoregressive structure based on observation of the underlying matrix. End-of-study differences in various measures of pain interference were modeled using a similar protocol but were based instead on generalized linear regression procedures (SAS PROC GENMOD) to accommodate the ordinal structure of the measures. Thus, the latter measures were regressed on a model including a baseline measure of the dependent variable, the dichotomous treatment proxy, visit, and an interaction term crossing treatment and visit using a log link under an assumed negative binomial distribution. Covariance structure was treated as first-order autoregressive. Tests of the study hypotheses were based on an end-of-study contrast (visit 6) of estimated LSM between the placebo and pregnenolone conditions. Outcomes of secondary measures were tested using similar protocols. Missing data were addressed using full information maximal likelihood model procedures.

### Neurosteroid Quantification by Gas Chromatography With Tandem Mass Spectrometry 

Serum samples of 1 mL were subjected to triplicate liquid-liquid extractions using ethyl acetate. Combined extracts were further purified by collecting fractions of interest from an Agilent 1100 Series high-performance liquid chromatography system (Agilent Technologies) equipped with a 25-cm × 4.6-mm × 5-μm LiChrosorb DIOL-5 high-performance liquid chromatography column (Merck KGaA) under normal phase conditions. Purified samples were subsequently derivatized with heptafluoroacetic anhydride and injected onto an Agilent 7013A gas chromatography–triple quadrupole mass spectrometer with chromatographic separations performed on the interfaced Agilent 7890B GC. A splitless injection of 2 μL was used on a 30.0-m × 0.25-mm × 0.25-μm installed HP-5MS GC column. Twenty percent of the injections were performed in duplicate. Multiple reaction monitoring transition states were optimized for precursor and product ions using the 2 most intense precursor-to-product ion transitions for each analyte. The more intense transition was used for target ion quantitation and the second one as the reference ion for structural confirmation (qualifier ion) and added specificity.

## Results

### Participants

The mean (SD) age of the 94 participants was 37.5 (9.8) years and 84 (89.4%) were male (Table 1). Fifty-three participants (56.4%) self-reported their race as Caucasian and 31 (33.0%) self-reported their race as African American. Participant demographic factors (age, sex, race) did not differ significantly by treatment condition following randomization. Mean (SD) pain diary and pain recall ratings at baseline were 5.03 (1.55) and 4.95 (1.59), respectively (0-10 numerical rating scale scale). Pain ratings were 8% higher in the pregnenolone condition at baseline, and there were differences in the mean level of pain interference between groups. No serious adverse events occurred during the trial, and pregnenolone was well tolerated. Adverse events were captured by the Hillside Adverse Events Scale, administered by interview at each study visit (eTable 1 in Supplement 2). Generally, few adverse events were reported and were relatively evenly distributed across both pregnenolone- and placebo-treated groups.

### Pain Intensity (Primary Outcome)

**Figure 2.**
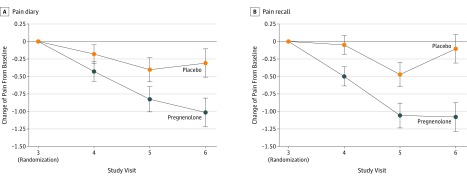
Changes in Low Back Pain by Treatment Group A, Pain intensity as recorded in a pain diary was significantly improved in the group randomized to pregnenolone compared with placebo at visit 6. B, Pain intensity according to recall was also significantly improved in the group randomized to pregnenolone compared to placebo at visit 6. Error bars indicate SE.

Baseline unadjusted mean (SE) pain diary scores in the placebo- and pregnenolone-treated groups were 4.83 (0.23) and 5.24 (0.22), respectively (baseline unadjusted mean [SE] ratings for pain recall were 4.78 [0.24] and 5.15 [0.23]) (Table 2). Unadjusted mean (SE) pain recall scores following treatment (visit 6) were 4.74 (0.26) for the placebo group and 4.19 (0.30) for the pregnenolone group. Unadjusted mean (SE) ratings for pain recall following treatment were 4.86 (0.27) for placebo and 4.18 (0.29) for pregnenolone. To test the primary hypothesis positing a pregnenolone-associated decrease in chronic low back pain, pain diary scores of low back pain intensity were regressed on a proxy variable for treatment status (pregnenolone or placebo), visit (visit 4, 5, or 6), and an interaction term crossing treatment with visit and controlling for baseline pain scores. Differences in pain intensity ratings between treatment conditions were tested for all postbaseline visits using contrasting model-derived LSM estimates (Table 3). Pain intensity ratings decreased progressively from baseline over the 4-week study period in pregnenolone-treated participants (Figure 2A), reaching a significant 12% decrease relative to placebo at visit 6 for pain diary ratings. The LSM (SE) difference in mean pain scores between treatment groups at the final study visit was −0.56 (0.25) (*P* = .02). Ratings of pain recall exhibited a similar but more pronounced pattern, decreasing significantly to approximately 15% below placebo at visit 6; the LSM (SE) difference was −0.70 (0.27) (*P* = .01) (Figure 2B). Odds ratios (ORs) for the probability of 20% or greater decrease in pain diary (OR, 2.62; 95% CI, 1.06-6.50; *P* = .04) and pain recall (OR, 2.68; 95% CI, 1.07-6.74; *P* = .04) ratings were more than 2.6 times greater in the pregnenolone-treated group than placebo-treated group (eTable 3 in Supplement 2). For pain diary intensity, 51.2% of those randomized to pregnenolone reported a 20% reduction in pain, while only 28.6% of those randomized to placebo reported a 20% reduction in pain. Results were similar for pain recall, as 48.8% of those receiving pregnenolone reported 20% or greater reduction in pain compared with 26.2% of those receiving placebo.

**Table 2.** Unadjusted Means and Difference Scores of Pain Diary and Pain Recall Ratings at Each Study Visit

Visit and Group	Pain Diary		Pain Recall	
	Patients, No.	Mean (SE)	Patients, No.	Mean (SE)
Visit 3				
Placebo	49	4.83 (0.23)	49	4.78 (0.24)
Pregnenolone	45	5.24 (0.22)	43	5.15 (0.23)
Difference		−0.40 (0.32)		−0.38 (0.33)
Visit 4				
Placebo	48	4.61 (0.29)	49	4.72 (0.28)
Pregnenolone	45	4.81 (0.24)	44	4.70 (0.24)
Difference		−0.19 (0.38)		0.02 (0.37)
Visit 5				
Placebo	47	4.56 (0.27)	47	4.44 (0.26)
Pregnenolone	44	4.43 (0.30)	44	4.23 (0.29)
Difference		0.14 (0.40)		0.21 (0.39)
Visit 6				
Placebo	42	4.74 (0.26)	42	4.86 (0.27)
Pregnenolone	41	4.19 (0.30)	41	4.18 (0.29)
Difference		0.55 (0.40)		0.68 (0.39)

**Table 3.** Differences From Baseline for Pain Intensity Scores Obtained From the Diary and From Recall

Visit and Group	Diary				Recall			
	Estimate (SE)	*df*	*t*	*P* Value	Estimate (SE)	*df*	*t*	*P* Value
Placebo at visit 4	4.89 (0.17)				4.94 (0.18)			
Pregnenolone at visit 4	4.65 (0.17)				4.60 (0.19)			
Difference	−0.24 (0.24)	169.5	−1.00	.32	−0.34 (0.26)	196.7	−1.33	.19
Placebo at visit 5	4.66 (0.17)				4.52 (0.18)			
Pregnenolone at visit 5	4.25 (0.17)				4.06 (0.19)			
Difference	−0.41 (0.24)	171.5	−1.70	.09	−0.46 (0.29)	198.8	−1.79	.07
Placebo at visit 6	4.67 (0.17)				4.79 (0.19)			
Pregnenolone at visit 6	4.11 (0.18)				4.09 (0.19)			
Difference	−0.56 (0.25)	180.80	−2.27	.02	−0.70 (0.27)	207	−2.59	.01

The magnitude of the effect of pregnenolone or placebo was explored within each group. Data scale mean change in pain diary scores were calculated including only those with scores at both baseline and visit 6. Participants receiving pregnenolone reported a change in pain rating of 1.01 (baseline mean [SE], 5.20 [0.23]; visit 6 mean [SE], 4.19 [0.30]), an improvement of approximately 20%. Individuals receiving placebo reported a data scale mean change of only 0.30 (baseline mean [SE], 5.04 [0.23] visit 6 mean [SE], 4.74 [0.26]), a 6% improvement in pain intensity rating.

### Pain Interference

In addition to pregnenolone-associated effects on pain intensity ratings, pregnenolone use was associated with significant amelioration of pain interference on 2 of 7 indices: activity (LSM [SE] change, 0.71 [0.11]; *P* = .03) and work (LSM [SE] change, 0.71 [0.12]; *P* = .04) (Table 4). In an artifact of randomization, all 7 pain interference measures were substantially elevated in the pregnenolone-treated condition relative to placebo at baseline, 4 of which were statistically significant. Despite higher baseline values in the pregnenolone-treated group, all but 2 of these pain interference measures decreased by more than 10% relative to placebo (sleep, walking) at visit 6, with 5 declining more than 20%. The lack of statistical significance may in part reflect Type II errors related to relatively small sample size.

**Table 4.** Differences in Pain Interference Domains at Study Completion by Treatment Group

Pain Interference at Visit 6	Estimate (SE)	*z* Value	*P* Value
Overall mean interference			
Placebo	2.21 (0.26)		
Pregnenolone	1.84 (0.23)		
Difference	0.83 (0.14)	−1.12	.26
Mood			
Placebo	2.18 (0.33)		
Pregnenolone	1.75 (0.29)		
Difference	0.80 (0.17)	−1.02	.31
Work			
Placebo	2.44 (0.28)		
Pregnenolone	1.74 (0.23)		
Difference	0.71 (0.12)	−2.05	.04
Sleep			
Placebo	2.39 (0.30)		
Pregnenolone	2.38 (0.31)		
Difference	0.99 (0.17)	−0.05	.96
Activity			
Placebo	2.88 (0.32)		
Pregnenolone	2.04 (0.26)		
Difference	0.71 (0.11)	−2.15	.03
Walking			
Placebo	1.79 (0.26)		
Pregnenolone	1.86 (0.25)		
Difference	1.03 (0.20)	0.17	.86
Relationships			
Placebo	1.45 (0.30)		
Pregnenolone	1.18 (0.20)		
Difference	0.82 (0.18)	−0.91	.36
Enjoyment			
Placebo	2.20 (0.26)		
Pregnenolone	1.85 (0.30)		
Difference	1.85 (0.30)	−0.99	.32

### Other Outcomes

Change in additional outcomes (sleep, depression, resilience, working memory, executive function, posttraumatic stress disorder, and physical function) between treatment conditions were tested for all postbaseline visits. There were no statistically significant treatment differences for any of these outcomes in this cohort with chronic low back pain (eTable 3 in Supplement 2).

### Neurosteroid Levels

Baseline serum levels of pregnenolone did not differ between groups (mean [SE], 585.26 [48.85] pg/mL for placebo vs 585.26 [48.85] pg/mL for pregnenolone; *P* = .99) (eTable 4 in Supplement 2). Serum pregnenolone levels more than doubled after 1 week of 100 mg of pregnenolone (mean [SE], 599.68 [54.70] pg/mL vs 1352.50 [107.57] pg/mL; *P* < .001), further increased after a week of 300 mg of pregnenolone (mean [SE], 684.04 [63.02] pg/mL vs 2443.89 [216.11] pg/mL; *P* < .001), and increased to a level almost 5 times greater than placebo after 2 weeks of 500 mg of pregnenolone (mean [SE], 616.73 [51.20] pg/mL vs 2786.24 [285.94] pg/mL; *P* < .001).

Allopregnanolone, a pregnenolone metabolite with analgesic properties, exhibited a pattern similar to pregnenolone. Allopregnanolone levels did not differ between groups at baseline (mean [SE], 62.22 [5.10] pg/mL for placebo and 67.96 [9.83] pg/mL for pregnenolone; *P* = .95); however, participants receiving pregnenolone had significantly increased allopregnanolone levels after 1 week of 100 mg of pregnenolone (6.5-fold increase; mean [SE], 59.73 [5.64] pg/mL vs 508.98 [55.31] pg/mL; *P* < .001). Allopregnanolone levels markedly increased again after participants received 1 week of 300 mg of pregnenolone (14.3-fold increase; mean [SE], 71.22 [11.07] pg/mL vs 1036.50 [108.04] pg/mL; *P* < .001) and continued to increase after 2 weeks of 500 mg (17.6-fold increase; mean [SE], 61.32 [6.00] pg/mL vs 1263.10 [136.90] pg/mL; *P* < .001). Changes in pregnanolone, another positive GABA_A_ receptor modulator, were similar, showing no difference at baseline (mean [SE], 32.75 [2.02] pg/mL for placebo vs 34.98 [25.19] pg/mL for pregnenolone; *P* = .98), but increasing significantly after 2 weeks of 500 mg of pregnenolone (mean [SE], 32.84 [2.28] pg/mL vs 883.87 [166.57] pg/mL; *P* < .001). Serum levels of androsterone, a neurosteroid that also positively modulates GABA_A_ receptors, did not differ at baseline between groups (mean [SE], 177.60 [10.92] pg/mL for placebo vs 171.67 [14.06] pg/mL for pregnenolone; *P* = .70) and decreased slightly in the pregnenolone group (mean [SE], 148.93 [11.07] pg/mL) compared with the placebo group (mean [SE], 186.26 [12.32] pg/mL) at visit 6 (*P* = .02).

## Discussion

Chronic low back pain is one of the most common diagnoses among Iraq- and Afghanistan-era veterans, and commonly used medications like opioids may lack effectiveness and increase risks of misuse, overdose, and death. The current study sought to determine whether a novel neurosteroid compound could safely and effectively reduce chronic low back pain symptoms in Iraq- and Afghanistan-era veterans. To our knowledge, this is the first phase 2 trial investigating the use of pregnenolone for a chronic pain condition. Results demonstrate that pregnenolone was safe and well tolerated. Compared with placebo, individuals randomized to pregnenolone reported significant improvement in both pain intensity and pain interference ratings.

Over the 4-week treatment with study medication, the LSM (SE) difference in mean pain scores between groups at the final study visit was −0.56 (0.25) (*P* = .02) from pain diary reports and −0.70 (0.27) (*P* = .01) from pain recall reports. In addition, pain interference scores improved in 2 domains, work (LSM [SE] change, 0.71 [0.12]; *P* = .04) and activity (LSM [SE] change, 0.71 [0.11]; *P* = .03). In order to explore the magnitude of effect within each group, we calculated data scale (raw mean) differences for each treatment group. The data scale mean change in pain diary scores (including only those with scores at both baseline and visit 6) reported by participants receiving pregnenolone was 1.01 (baseline mean [SE], 5.20 [0.23]; visit 6 mean [SE], 4.19 [0.30]), an improvement of approximately 20%, which is consistent with the Initiative on Methods, Measurement, and Pain Assessment in Clinical Trials (IMMPACT) definition of a minimal clinically important difference.^27,28^ It is additionally important to consider that further reductions in pain intensity may have resulted if participants had received more than 4 weeks of study medication, as pain ratings improved substantially between visits 5 and 6 (Figure 2). Moreover, it is also possible that our final dose of pregnenolone (500 mg/d for 2 weeks) was not high enough to produce maximal pain relief. The effects of treatment duration and dose need to be explored in future studies.

Individuals receiving placebo reported a data scale mean change of only 0.30 (baseline mean [SE], 5.04 [0.23]; visit 6 mean [SE], 4.74 [0.26]), a 6% improvement in pain intensity rating. The substantial response difference between those receiving pregnenolone (20%) or placebo (6%) is notable, as the likelihood of placebo response to pharmacological pain treatments can be considerably higher, sometimes higher than the intervention itself.

Another indicator of the magnitude of pregnenolone’s effects is by responder analysis. Individuals randomized to pregnenolone were 2.6 times more likely to report a reduction in pain intensity ratings of 20% or greater by both pain diary (OR, 2.62; 95% CI, 1.06-6.50; *P* = .04) and pain recall (OR, 2.68; 95% CI, 1.07-6.74; *P* = .04) (eTable 2 in Supplement 2). Other key considerations in determining a drug’s clinical significance are tolerability and the amount of time it takes to achieve the intended effect. In the current study, participants reported significant improvements in pain intensity and interference scores after only 4 weeks of pregnenolone treatment. Additionally, pregnenolone was well tolerated, with a favorable adverse effect profile. Taken together, in addition to a statistically significant reduction in pain intensity and interference scores, these factors suggest that pregnenolone treatment resulted in safe and clinically meaningful improvements in chronic low back pain.

A relatively unique quality of neurosteroids is the potential to be both a biomarker and a therapeutic intervention. Several preclinical^11,29,30,31,32,33,34^ and clinical^25,26^ studies provide strong evidence for the potential role for allopregnanolone as a biomarker of pain and analgesia. In the current study, pregnenolone administration resulted in decreases in pain intensity and interference scores, and increased serum levels of both pregnenolone and allopregnanolone after treatment. Administration of adjunctive pregnenolone may thus produce analgesic effects via enhancement or restoration of depleted allopregnanolone and/or pregnenolone levels.

### Strengths and Limitations

Strengths of this investigation include the prospective, randomized, double-blind, placebo-controlled design. In addition, retention rate was high, as 83% of participants randomized completed the final study visit.

This study also had limitations, including the relatively short duration of time that participants received study medication, as pain scores may have continued to improve with longer dosing duration. Additionally, we focused on chronic low back pain, and these findings may not generalize to other types of pain. Furthermore, although our findings may provide initial data on effective therapeutic dose, this study was neither designed nor powered to determine dose response; pregnenolone was increased at a fixed, escalating dose primarily for tolerability purposes and not to determine an effective optimal dose.

## Conclusions

Pregnenolone treatment for 4 weeks was associated with clinically meaningful and statistically significant improvements in pain intensity ratings compared with placebo. Pregnenolone was well tolerated in this study, exhibiting promise as a safe, adjunctive treatment for chronic low back pain.

## Notes

Supplement 1. **Trial Protocol**

Supplement 2. **eTable 1.** Adverse Events as Reported on Hillside Adverse Event Scale **eTable 2.** Odds Ratios for 20% Improvement of Pain Based on Treatment with Pregnenolone vs Placebo **eTable 3.** Secondary Outcomes **eTable 4.** Neurosteroid Level Changes by Treatment Group

Supplement 3. **Data Sharing Statement**
