# Supplementary material for: Effect of Pregnenolone vs Placebo on Self-reported Chronic Low Back Pain Among US Military Veterans: A Randomized Clinical Trial
Source: JAMA Netw Open. 2020 Mar 2;3(3):e200287. doi: 10.1001/jamanetworkopen.2020.0287 (PMC7052727; doi:10.1001/jamanetworkopen.2020.0287)
Supplement: Supplement 1. — Trial Protocol [file jamanetwopen-3-e200287-s001.pdf]

PROJECT OVERVIEW

Neurosteroids as Novel Therapeutic Agents for Chronic Pain in OEF/OIF Veterans

PURPOSE

More than 1.64 million military personnel have served during Operation Enduring Freedom / Operation Iraqi Freedom (OEF/OIF) to date (Rand Report: Invisible Wounds of War), and the majority of these Veterans report a myriad of chronic pain symptoms (Office of Public Health and Environmental Hazards, 2010). Chronic pain adversely affects an individual's emotional and physical well-being, cognition, quality of life, and functional abilities, and increases suicide risk (Pain Task Force, 2009). Chronic pain also represents a multi-billion dollar annual expense for the Veteran's Healthcare System (Bilmes, 2007). Additionally, chronic pain disorders can be challenging to treat in OEF/OIF Veterans, as this cohort often reports complex co-occurring psychological and physical symptoms (Taylor et al., 2012; Helmer et al., 2009; Gironda et al., 2009). Moreover, a higher percentage of wounded military personnel are fortunately now surviving wounds that in prior wars would have been fatal (Gawande, 2004; Hoge et al.,2004), but suffering from persisting pain conditions. The development of new therapeutics for the reduction and alleviation of pain symptoms in OEF/OIF Veterans thus represents an acute and critical unmet need. Although non-pharmacological interventions are often very helpful for the management of chronic pain symptoms, the addition of adjunctive pharmacological strategies could optimize the effects of both approaches, potentially resulting in synergistic effects and enhanced reduction of pain symptoms. However, current pharmacologic interventions for chronic pain are suboptimal and frequently accompanied by unwanted side effects, and new therapeutics are thus urgently needed.

Neurosteroids are endogenous molecules that are enriched in human brain and represent promise for pain therapeutics. Extensive data in rodent models demonstrates that neurosteroids exhibit pronounced analgesic actions, and our data in OEF/OIF Veterans suggests that neurosteroids are decreased in the setting of pain symptoms in clinical populations (Kilts et al., 2010). Neurosteroids are hence logical candidates for pain alleviation that have not been tested yet for this indication, despite compelling preclinical and clinical evidence. Moreover, a number of these molecules are available over-the-counter in the U.S. as dietary supplements, and they hence are immediately translatable into clinical trials investigating the potential of these novel approaches for the amelioration of persistent pain symptoms – i.e. via a strategy of supplementation of endogenously produced compounds that demonstrate analgesic actions.

RESEARCH DESIGN AND METHODS

The proposed randomized controlled trial (RCT) will investigate adjunctive pregnenolone as a potential analgesic in OEF/OIF Veterans with chronic low back pain. This will be a 4-week, randomized, double-blind placebo-controlled pilot study preceded by a one-week placebo lead-in period (all participants). Proposed sample size estimates are 45 patients per group (it is estimated that up to 120 Veterans will be randomized in order to obtain the sample size of 45 participants per group).

Experimental Design: The proposed design is a randomized, double-blind, two-arm trial of pregnenolone and placebo to determine the possible analgesic effects in OEF/OIF Veterans with chronic low back pain. The total study duration is 8 weeks (6 study visits followed by two-follow up phone calls). All patients will monitor pain symptoms for one week with pain diaries, followed by a 1-week placebo-only lead-in period, 90 subjects will be randomly assigned to one of two groups. Of these subjects, 45 subjects will receive pregnenolone, and 45 subjects will receive placebo for 4 weeks (in addition to the taper period of 5 days). Patient interview assessments and laboratory studies will be performed at each interview time point. Pregnenolone, allopregnanolone and other neurosteroid metabolites will be determined by gas chromatography / mass spectrometry (GC/MS), proceeded by high performance liquid chromatography (HPLC). Those subjects randomized to pregnenolone will receive this neurosteroid on the following schedule:

--Pain symptom monitoring with pain diaries for 1 week, then
--Placebo lead-in phase 0 mg in two doses (0 mg, PO, BID) for 1 week, then
--Pregnenolone 100 mg in divided doses (50 mg, PO, BID) for 1 week, then
--Pregnenolone 300 mg in divided doses (150 mg, PO, BID) for 1 week, then
--Pregnenolone 500 mg in divided doses (250 mg, PO, BID) for 2 weeks.

Dosing: Pregnenolone fixed escalating up to 500mg/day will consist of the following schedule:
Visit 1 (week 0, screening visit) and pain symptom assessment
Visit 2 (week 1) placebo lead-in (all participants)
Visit 3 (week 2, baseline visit): 50mg PO, BID x 1 week,
Visit 4 (week 3): 150mg PO, BID x 1 week,
Visit 5 (week 4): 250mg PO, BID for two weeks.
Visit 6 (week 6): No medication will be dispensed; study medication will be tapered by 100mg per day and then discontinued. The FDA has reviewed this protocol and issued an Investigational New Drug number (IND #114,799) for this randomized controlled trial utilizing the pregnenolone doses above for low back pain, and has provided a formal permission to proceed letter for this study. In addition, Dr. Christine Marx (the PI's primary mentor on this VA funded Career Development Award) currently holds FDA IND numbers for the use of pregnenolone for treatment of schizophrenia (IND #71,768), PTSD (IND #73,099) and TBI (IND #78,270).

Methodology for Neurosteroid Identification and Quantification:

Neurosteroid quantification will be performed by highly sensitive and specific gas chromatography/mass spectrometry (GC/MS), preceded by high performance liquid chromatography (HPLC) purification as previously described (Marx et al. 2006a, Marx et al 2006b), with modifications. HPLC purification will be performed on an 1100 Series Agilent instrument. Standards and samples will be derivatized utilizing heptafluorobutyric acid anhydride (HFBA) and injected onto an Agilent 5973 mass spectrometer (MS) coupled to an Agilent 6890 N gas chromatograph (GC) equipped with an Agilent HP-5MS 30 m×0.250 mm×0.25 μm capillary column. Positive ion electron impact (EI) ionization will be utilized in the GC/MS component. Serum samples will be analyzed in duplicate in positive ion electron impact mode (EI) using helium as the carrier gas. In addition to the GC/MS retention time characteristic of each neurosteroid, the definitive structural identification of each neurosteroid will be provided by its unique mass fragmentation pattern. Mass spectrometer single ion monitoring (SIM) will be used to focus on the most abundant ion fragment for each HFBA derivative (pregnenolone 298.2, allopregnanolone 496.2, pregnanolone 496.2, and androsterone 486.2). Only peaks with a signal to noise ratio greater or equal to 5:1 are integrated. The limit of neurosteroid quantification with this methodology is 2 picograms for pregnenolone, allopregnanolone, androsterone, and pregnanolone (femtomolar sensitivity). Intra-assay coefficients of variation continue to be very good utilizing this approach. For example, our last two serum sample investigations produced intra-assay coefficients of variation that were 1.8% and 1.6% for pregnenolone, 3.7% and 3.9% for allopregnanolone, 4.0% and 3.9% for androsterone, and 2.0% and 2.9 % for pregnenolone. These intra-assay coefficients of variation are in the same range as our prior investigations utilizing an EI approach.

Pharmacogenetics

We propose to conduct an exploratory analysis to assess the potential influence of genetic variations in enzymes involved in neurosteroid synthesis (P450scc, 3β-HSD, 5α-reductase, 3α-HSD) on serum concentrations of neurosteroids and pregnenolone treatment response. Common variations in the genes coding

for these enzymes (*CYP11A1*, *HSD3B1*, *HSD3B2*, *SRD5A1*) that influence enzyme activity have been identified (Pusalkar M 2009, Park et al 2007, Shimodaira et al 2010, Takahashi et al 2009).

Blood samples for pharmacogenetics will be collected in one EDTA (purple top) vacutainer from each study patient at the baseline visit. All blood samples will be coded and stored in a -80 degree freezer at the Durham VAMC. When the study is closed to enrollment (end of Year 3, beginning of Year 4), all coded frozen blood samples will be transferred to Duke University's Center for Human Genetics for genotyping. SNP genotyping will be performed on Applied Biosystems' 7900HT Real-Time PCR System using both pre-developed and custom TaqMan® SNP Genotyping Assays.

The availability of potential neurosteroid biomarkers in serum combined with possible candidate gene data could lead to "population segmentation" approaches to optimal clinical care in patients with chronic pain receiving pregnenolone, providing the "right drug for the right patient" at the "right dose" (Breier 2005). These projects could also lead to the characterization of serum neurosteroid biomarkers and/or candidate genes that identify Veterans with chronic pain and comorbid disorders who are most likely to respond to an intervention with pregnenolone or other neurosteroid agents.

RISK/BENEFIT ASSESSMENT

Potential Risks

a) Physical Risk: Physical risks associated with participation in this study include the risks of drawing blood and adverse effects from the study medication. Possible side effects from drawing blood include bruising, bleeding or pain at the injection site, and (rarely) fainting and infection. Blood draws at each visit are minimal risk. In terms of the study medication, pregnenolone has been well-tolerated at doses up to 700 mg per day. Side effects exceeding placebo that have occurred in previous clinical trials with pregnenolone conducted by Dr. Chris Marx, the applicant's primary mentor, have included mild restlessness, mild cold extremities, mild muscle pain/stiffness, and mild headache. If a subject develops side effects at any dose, they may be brought down to the previously tolerated dose or they may be withdrawn from the study and, as appropriate, referred for evaluation and treatment. We will perform laboratories at each study visit (Chem 7, CBC, GI Panel) and an ECG at study entry and completion. No serious adverse events have been reported to date utilizing pregnenolone (sold as a dietary supplement over-the-counter in the U.S.).

Of note, subjects will NOT be tapered from their current stable medication regimen; pregnenolone will only be "added on" to treatment-as-usual.

b) Emotional Distress: Subjects may potentially experience emotional distress as a result of participating in this study. The study procedures could potentially lead to emotional distress from discussing trauma histories during mental health assessments. Each subject will be closely monitored for emotional distress, suicidal ideation and homicidal ideation at screening and each study visit. If a subject becomes significantly distressed or their psychiatric symptoms worsen and intervention is deemed appropriate, they will be walked to the emergency psychiatry clinic (on site) for evaluation and treatment (including potential hospitalization). The study medication will be discontinued and they will be withdrawn from the study if the patient is hospitalized, or if medical and/or psychiatric issues take precedence over study participation.

c) Safety Plan: We will implement a rigorous safety plan that encompasses close monitoring and care of medical issues in this study. The conduction of safety monitoring includes the following procedures outlined in

the Physician On-Call Cascade (please see below in Adverse Event and Safety Evaluation section). This study would be conducted under FDA IND # 114,799. We will follow all FDA guidelines for annual progress, adverse event, and other reporting procedures for this outpatient randomized control trial.

Adequacy of Protection from Risks

Physical Risk and Subject Safety Possible side effects from drawing blood (as mentioned above) will be attended to as usual in the phlebotomy laboratory. If a subject experiences serious adverse effects from the study medication, it will be discontinued and s/he will be withdrawn from the study. The subject will immediately be referred for appropriate evaluation and treatment. All serious adverse effects will be documented and reported as required by the FDA and local IRB committee.

Emotional Distress and Subject Safety All subjects will be carefully assessed before the study and will be made aware of emergency services. In addition, they will be closely monitored at screening and each study visit. During the informed consent process, they will be advised that the study procedures could potentially lead to distress and that they may withdraw from the study at any time without adversely affecting their medical care or any benefits they may be receiving. If a subject withdraws from the study and they are not currently in psychiatric or medical treatment, they will be referred to the appropriate mental health clinic (MHC, PTSD clinic, etc.) and/or primary care provider. If a subject is significantly distressed during a study visit and intervention is deemed appropriate; they will be walked to the emergency psychiatry clinic onsite for evaluation and treatment (including potential hospitalization if clinically indicated). Dr. Naylor and Dr. Marx (and the physician study team) will be immediately available by cell phone at all times for any concerns regarding potential worsening psychiatric symptoms, including suicidal and homicidal ideation. In terms of risks to confidentiality, pertinent information regarding potential harm, including suicidal and homicidal intent will be shared as necessary and required by law with clinicians and/or the appropriate authorities. In such circumstances, records may be made available to authorities, even without the subject's consent. Upon completion of the study any subjects not currently in psychiatric treatment will be referred for treatment as deemed appropriate.

Potential Benefit of the Proposed Research to the Subject and Others: While study participants may not receive benefits from the proposed research other than monetary compensation ($75 per visit), their participation may lead to a better understanding of their symptomatology. For those not currently treated, study participation may lead to referral for treatment upon completion (or withdrawal) of the study. In terms of benefit to others, knowledge gained from the study may help the evaluation and treatment of patients with chronic pain disorders and common comorbidities in the future. No serious adverse events have been attributed to pregnenolone to date.

SUBJECT RECRUITMENT

Up to 120 OEF/OIF Veterans with moderate to severe chronic low back pain between the ages of 18-65 will be enrolled in order to obtain the 90 subjects per randomization arm (45 pregnenolone/45 placebo) Both male and female subjects and all ethnic groups will be eligible to participate in this study. All subjects will be currently experiencing chronic low back pain, as evidenced by prior medical documentation and weekly mean of 24-hour average pain score ≥ 4 (Numerical Pain Rating Scale, scale 0-11) at baseline. Subjects will be recruited from a variety of sources including community based PCPs, VA clinics, Vet Centers, the Durham VA Medical Center and VISN-6 VA Mid-Atlantic MIRECC Registry of Post-deployment Health in OEF/OIF Veterans. Information about the study may be disseminated through a variety of conduits and outreach efforts including brochures, flyers, MIRECC website, VA mailings and newsletters, radio, TV, university/college campuses, newspaper ads,

and educational presentations to health care professionals. All advertisements will be approved by the Durham VA IRB prior to use.

OEF/OIF/OND-era Veterans will be recruited from the MIRECC Repository. To recruit Veterans from the MIRECC Repository, the current study's IRB-approved recruitment letter will only be sent to subjects who have agreed (in the ICF) as part of their data collection for the VISN 6 MIRECC Study of Post-Deployment Mental Health (IRB # 0933) and Post-Deployment Mental Health Data Repository (IRB # 01706) to be re-contacted for future research studies. In order to target Veterans from the repository who meet our inclusion/exclusion criteria we will request that the VISN 6 MIRECC provide the names, phone numbers, and mailing address of Veterans who meet the following inclusion/exclusion criteria:

- Are OEF/OIF/OND Veterans;
- Report low back pain symptoms;
- Are age 65 or younger;
- Do not meet criteria for Bipolar Disorder (current or lifetime);
- Do not meet criteria for Schizophrenia (current or lifetime)
- Do not meet criteria for a Psychotic Disorder (current or lifetime);

<u>Recruitment Procedures</u>
Potential participants will be contacted initially via U.S. mail with a signed and IRB-approved recruitment letter. For individuals who have been sent a letter, a follow-up telephone call will take place to confirm receipt of letter, to inquire if a participant may be interested in study participation, and to pre-screen for study qualification (via IRB telephone script entitled "follow-up letter"). If potential participants initiate contact with the study team by telephone (i.e. if participants are responding to an IRB-approved advertisement or have been referred to the study by another healthcare professional and thus did not receive an IRB-approved recruitment letter), individuals will be pre-screened for study qualification using the IRB-approved telephone script entitled "call from patient." Participants who meet pre-qualification criteria will be invited to schedule an appointment at the Durham VAMC to complete a screening visit, in which they will learn details about study involvement and will be asked to provide informed consent. Participants who attend this visit will be compensated for their time, whether or not they decide to participate further in the study.

<u>SELECTION OF SUBJECTS</u>
<u>Inclusion Criteria</u>:
1. OEF/OIF Veterans, 18-65 years of age, with chronic low back pain.
2. Based on medical history and medical records, have low back pain (Thoracic Vertebrae 6 or below) present on most days for the preceding 6 months or longer, and fulfill all disease diagnostic criteria (please see disease diagnostic criteria below).
3. Have a weekly mean of 24-hour average pain score ≥ 4 at baseline.
4. Negative pregnancy test if female. Sexually active subjects are required to use a medically acceptable form of birth control if they are of childbearing potential and could become pregnant during the study. A medically acceptable form of birth control includes non-hormonal intrauterine devices, surgical sterilization, or double barrier methods (e.g., diaphragm with contraceptive jelly, condom with contraceptive foam, cervical caps with contraceptive jelly). Sexual abstinence with agreement to continue abstinence or to use a medically acceptable method of contraception (as listed above) should sexual activity occur is permissible.
5. No change in medications less than 4 weeks before baseline.
6. No anticipated need to alter psychotropic or pain medications for the 6-wk study duration (as determined by study physician's review of records and/or discussion with prescribing physician).

7. Ability to fully participate in the informed consent process.

Exclusion Criteria:
1. Unstable medical or neurological illness, including seizures, renal impairment, cerebral vascular accident or prostate, uterine or breast cancer (since pregnenolone supplementation could theoretically increase downstream steroid metabolites).
2. Use of oral contraceptives or other hormonal supplements.
3. Significant suicidal or homicidal ideation requiring intervention.
4. Daily use of long or short-acting narcotic medications.
5. Current DSM-IV diagnosis of bipolar disorder, schizophrenia, or other psychotic disorder, or cognitive disorder due to a general medical condition (other than if related to mild TBI).
6. Female patients who are pregnant or breast-feeding.
7. Known allergy to study medication.
8. History of moderate or severe TBI (mild TBI is permissible).
9. DSM-IV criteria met for alcohol and/or other substance abuse or dependence within past three months (excludes caffeine and nicotine).
10. Have received epidural steroids, facet block, nerve block, or other invasive procedures aimed to reduce low back pain within the past 3 months prior to Visit 1.
11. Completion of daily diaries for less than 70% of days between Visit 1 and Visit 2 and between Visit 2 and Visit 3.
12. Have ongoing or anticipated disability compensation or litigation issues, in the best judgment of the investigator.
13. Have a presence of any factors/conditions, medical or other, that in the judgment of the investigator may interfere with performance of study outcome measures, such as treatment-refractory history.
14. Have serious or unstable cardiovascular, hepatic, renal, metabolic, respiratory, or hematologic illness, symptomatic peripheral vascular disease, or other medical condition or psychiatric conditions that, in the opinion of investigator and study physician, would compromise participation or be likely to lead to hospitalization during the course of the study.
15. Are non-ambulatory or require the use of crutches or a walker.
16. Current suicidal or homicidal ideation necessitating clinical intervention or representing an imminent concern, or history of suicide attempt in the past 3 years.

Disease Diagnostic Criteria

Patients must have clinical diagnosis of chronic low back pain. Pain must be present on most days for at least 6 months. The pain must be either restricted to low back or associated with radiation to the proximal portion of the lower limb only (Class 1 and 2 per Quebec Task Force on Spinal Disorders). Study candidates must not have: a) neurological radicular signs; b) presumptive compression of a spinal nerve root on a simple radiogram (that is, spinal instability or fracture); or c) compression of a spinal nerve root confirmed by specific imaging techniques (computerized tomography [CT], myelography, or magnetic resonance imaging [MRI]) or other diagnostic techniques (for example, electromyography). The above diagnosis shall be made by a study physician and will be based on history, and physical and neurological examination. In addition, absence of spinal fracture, spondyloisthesis grade 3 or 4, tumor, abscess or acute pathology in the low back/abdominal regional must be confirmed by historical record of imaging studies (any of the following: plain x-ray, CT, or MRI). As per the study physician’s opinion, there must be no clinically significant change in the condition since the time of imaging. A study physician will review medical charts and interview patients to determine disease criteria.

Pregnenolone: Pregnenolone is available over-the-counter and sold as a dietary supplement in the U.S., and estimated to be used by thousands of people. It has a very favorable side effect profile among clinical studies conducted to date; please see Appendix 3 for recent pregnenolone safety data summary. Despite its ubiquitous availability as a dietary supplement, pharmacokinetic data for pregnenolone are limited. Please see also summary of pregnenolone in the PDR for Nutritional Supplements (attached). No serious adverse events have been attributed to pregnenolone to date.

Treatment Compliance

Compliance for each visit interval is defined as taking between 80% and 120% of the study drug prescribed for that interval. For subjects who demonstrate noncompliance, the PI and/or study coordinator will counsel patients on the importance of study drug compliance and drug accountability. Patients who miss more than 6 consecutive doses of medication or are less than 80% compliant will be withdrawn from the study.

The following procedures will be employed to assure appropriate drug accountability:
- Drug accountability will be emphasized at the screening visit and throughout the trial.
- Drug accountability forms will be provided in the clinical trial records binder or similar file.
- Drug accountability will be monitored throughout the study.

Each patient will be instructed to return all study drug packaging and unused at each visit. Records will be kept of all drug dispensed to and returned by the subjects throughout the study.

CONSENT PROCESS

As outlined above, we are requesting a waiver of Informed Consent and HIPAA Authorization to identify and contact Veterans and MIRECC Repository participants who may be eligible for the study. A subject who is judged likely to meet all of the inclusion criteria and none of the exclusion criteria will meet with a member of the research team to discuss the research protocol, and to determine if the patient is capable of providing informed consent. This study utilizes an informed consent form. The subject will be provided with a description (verbal and written) of the informed consent form, which includes the risks along with procedures to minimize these risks, and the subjects' rights and responsibilities. Subjects will be given the opportunity to read the consent form and ask questions. Subjects will be assured that participation in this research study is voluntary and that they may withdraw from the study at any time without adversely affecting their medical care or any benefits they might be receiving. They may also refuse to answer any research questions during interviews. Subjects who are eligible for the study and choose to participate will sign the consent form in the presence of a member of the research team and a witness.

STUDY INTERVENTIONS

Primary Outcome Measures

- The primary efficacy outcome measure will be the weekly mean of the 24-hour average pain severity scores recorded daily on an 11-point Likert scale, an ordinal scale ranging from 0 (no pain) to 10 (worst possible pain). Patients will be asked to complete their pain diary at bed time.

Secondary Outcome Measures

- The first secondary outcome measure will be the Brief Pain Inventory (BPI) (Severity and Interference scores). The BPI is a self-reported scale that measures the severity of pain and the interference of pain on function. The scores range from 0 (no pain) to 10 (pain as severe as you can imagine). There are 4 questions assessing worst pain, least pain, average pain in the past 24 hours, and the pain right now (As described above average pain scores will be used in the primary outcome measure). The Interference scores range from

0 (does not interfere) to 10 (completely interferes). There are 7 questions assessing the interference of pain in the past 24 hours for general activity, mood, walking ability, normal work, relations with other people, sleep, and enjoyment of life. Questions from the BPI will be asked at each patient visit.

- The Tower of London is a clinician administered subtest from the BAC-A which measures executive functioning.
- Digit Sequencing Task is a clinician administered subtest from the BAC-A which measures working memory.
- The Davidson Trauma scale is a self-report questionnaire that measures trauma symptoms.

Routine laboratory assessments will be completed at each visit (CBC, Chem 7, GI panel) and will require the patient to donate approximately 10ml of blood. Additional blood samples will be collected for serum analysis during all study visits. Serum samples will be frozen in a -80 degree freezer. Upon completion of the study, samples will be thawed and analyzed using Gas Chromatography/Mass Spectrometry. Patients will receive an ECG at Visit 1 and the final study visit. Vital signs will be assessed and recorded at each study visit. Additional assessment measure descriptions are listed below.

***Study Timeline*:**

1. Referred patients and patients responding to IRB-approved advertisements or IRB-approved recruitment letters, phone calls (with IRB-approved phone script), will meet with a member of the research team to discuss the study and the risks and benefits of participation. In addition, patients will be screened for exclusion and inclusion criteria. If subjects are interested, the informed consent document will be discussed with them. Patients will have the option of taking the informed consent with them and discussing the matter with family, friends or clinicians. If the patient is followed by a psychiatrist, the psychiatrist will be consulted prior to randomization regarding the patient's appropriateness for enrollment in the study.
2. Once the informed consent is signed, the subject will proceed to the initial screening procedures. First, patients will receive a more detailed assessment than what was queried on the telephone pre-screening interview to determine whether they meet qualifications to participate in the study.
3. We will then determine whether subjects are in good health. A research physician will examine patients before starting the study. Patients will have a venous puncture for laboratory testing (please see Schedule of Events). Subjects will receive an ECG at Visits #1 and #6. Since patients may be on antidepressant and occasionally on antipsychotic medicines (treatment-as-usual), it is part of routine clinical care to receive an ECG at least once each year on these agents. A follow-up ECG at Visit #6 will be performed to confirm that there are no changes in ECG tracings (since there is one report of palpitations in the existing scientific literature following pregnenolone). Patients with significant abnormal physical exam, blood tests, or ECG will be excluded from the study and referred to their primary care provider.

Table 1. Study Schedule

Visit	1	2	3	4	5	6	Follow Up	Follow Up
	Screen	Baseline					Phone	Phone
**Week**	**0**	**1**	**2**	**3**	**4**	**6**	**7**	**8**
Inclusion/Exclusion (5 min)	X							
Informed Consent (15 min)	X							
Demographics (10 min)	X							
Medical History (10 min)	X							
Psychiatric Diagnosis (MINI) (15 min)	X							
Physical Exam and ECG (30 min)	X					X		
Vital Signs (5 min)	X	X	X	X	X	X		
Urine Collection: (5 min)								
Drug Screen	X	X		X		X		
Urinalysis	X	X	X	X	X	X		
Urine Pregnancy Test (females only)	X	X	X	X	X	X		
Concomitant Medications (5 min)	X	X	X	X	X	X		
Patient Pain Diary (5 min)	X	X	X	X	X	X		
Adverse Events (5 min)		X	X	X	X	X	X	X
Brief Pain Inventory (10 min)	X	X	X	X	X	X		
Patient's Global Impressions of Improvement (2 min)			X	X	X	X		
Fagerstrom Test for Nicotine Dependence (2 min)	X	X				X		
Beck Depression Inventory (5 min)	X	X	X	X	X	X		
Davidson Trauma Scale (7 min)	X	X	X	X	X	X		
Athens Insomnia Scale (5 min)	X	X	X	X	X	X		
Roland Morris Scale (5 min)	X	X				X		
BAC-A subtests (Tower of London and Digit Sequencing Task) (10 min)	X	X	X	X	X	X		
Connor Davidson Resilience Scale (5 min)	X	X	X	X	X	X		
36-item Short-Form Health Survey (5 min)	X	X	X	X	X	X		
Columbia Suicide Severity Rating Scale (5 min)	X	X	X	X	X	X	X	X
BLOOD DRAW (15 minutes)								
Blood draw (Pax Gene tube)	X							
Blood draw (2 purple top tubes)	X							
Blood draw (neurosteroids)	X	X	X	X	X	X		
Blood draw (TSH, prolactin)	X					X		
Blood draw (Chem 7, GI Panel, CBC)	X	X	X	X	X	X		
Dispense Drug or Placebo	N/A; Diary start	Placebo	Placebo or 50 mg Preg BID	Placebo or 150 mg Preg BID	Placebo or 250 mg Preg BID	N/A; taper begins	N/A; follow up phone call	N/A; follow up phone call
Total Time Commitment	179	99	94	94	94	126	10	10

4. Following the screening process, patients will begin a one-week period in which they will record pain levels in a daily diary (Visit #1). The following week (Visit #2), they will begin a one-week placebo-only lead-in period. On the days that patients are given study medication, a set of vital signs will be assessed including pulse, blood pressure, respiratory rate, temperature, and weight. Female participants will be asked to provide a urine specimen for a urine pregnancy test at point of care, during each study visit. With the study medication, the patients will be told that they could be getting placebo (a sugar pill) or active compound.

They will be asked to take the medication twice a day; once in the morning and once at night. The initial dose at Visit #2 will always be placebo (one-week placebo single-blind lead-in phase).

5. Subjects will return for a study visit every week (except for Week 5). Vital signs will be assessed at each visit. A member of the research team will ask subjects about possible side effects and adverse reactions (Adverse Event Form). Patients will be asked to return capsules of pregnenolone or placebo to determine compliance with study medication. The subject will then be given a new supply of capsules (either pregnenolone or placebo, depending upon random assignment) at Visits #3, #4, and #5. At baseline (Visit #1) and at the final visit (Visit #6), patients will receive a Chem 7, GI panel, CBC, TSH, prolactin, lipid panel, urine drug screen, and urinalysis; all patients will receive urinalysis and female patients will also receive a pregnancy test at all visits. Urine drug screens will be administered at Visits 1, 2, 4 and 6. A Chem 7, GI panel, and CBC will also be performed at Visits #2,3,4, and 5 (a Chem 7, GI panel, and CBC will therefore be performed at each study visit).
6. At Visits #1, 2, 3, 4, 5 and 6, three extra red-top tubes will be collected for determination of pregnenolone levels and pregnenolone metabolite levels. Serum will also be utilized for protein and other small molecule characterizations. At the end of these analyses, any remaining serum will be destroyed.
7. At Visit #1, two purple-top tubes and one PAX gene tube (blood RNA tube) will be collected for genetic analyses examining polymorphisms in the enzymes leading to neurosteroid synthesis and metabolism, as well as other candidate genes relevant to pathophysiology and treatment.
8. At Visit #6, patients will be instructed to taper the study medication by 100mg/day (for example, if the patient is taking 500mg of pregnenolone at Visit #6, the following day he will take 400 mg, the day after 300mg, etc. until the taper is complete).
9. At the final visit (Visit #6), patients will receive psychiatric assessments, vital signs, and side effect assessment. In addition, the physical exam and ECG will be repeated.
10. BPI, BDI, DTS, AIS, 36-item Short form Health Survey, selected BAC-A subtests, CDRS and CSSRS will be administered during each study visit. The Patient's Global Impressions of Improvement will be administered during Visits #3, #4, #5 and #6. The Roland Morris Scale and Fagerstrom Test for Nicotine Dependence **(**FTND) will be administered at Visit #1, #2 and #6. Please see schedule of events.
11. All subjects who complete the entire study (6 visits total) will receive two follow-up phone calls during Weeks #7 and #8 and be asked about adverse events (Hillside Adverse Events) and suicidality (CSSRS).

<u>ADVERSE EVENTS AND SAFETY EVALUATIONS</u>

<u>Adverse Events:</u> Adverse events will be recorded by administration of the Hillside Adverse Event Form and reviewed by a study physician (please see physician study team schedule/cascade). Adverse events will be assessed at all study visits after the screening visit (i.e. Visits 2-6). In addition, adverse events will be assessed by a follow-up telephone call at the end of the study medication taper (Week 7) and again one week after study medication has been stopped (Week 8). In addition to receiving these regular adverse effect assessments, patients will be given a toll free study number that they can call if they have questions regarding the dose escalation schedule or medication side effects. They will also be provided with the cell phone number of the study PI, Dr. Naylor, PhD as well as Dr. Marx, MD. Other covering VA physicians (as part of the call cascade) will be accessible to Dr. Naylor as per the call schedule.

Suicidality Safety Procedures for Follow-Up Phone Calls:
If a participant reports suicidality (suicidal ideation, gesture, or attempt) as per the Columbia Suicide Severity Rating (CSSRS, which is an FDA-recommended assessment for suicidality in clinical trials) during any follow-up phone call, a study physician will be notified immediately to determine whether the situation requires immediate clinical intervention. If a participant is thought be at imminent risk to self, he/she will be referred immediately to emergency psychiatric care at the Durham VA Emergency Department, to Psychiatric

Emergency Services (PEC) at the Durham VA, or to the participant's local Emergency Department for further evaluation. If the participant is unwilling or unable to get to emergency psychiatric care and appears to be in imminent danger of self-harm, the study team will contact local law enforcement who will follow up with the participant at their current location.

All adverse events will be reported to the Durham VA IRB and FDA as per institutional requirements. We will adhere to all FDA reporting guidelines for this study; the PI will be responsible for reporting any unexpected fatal or life-threatening adverse experience associated with use of the drug by telephone or fax no later than 7 calendar days after initial receipt of the information, submitting annual progress reports, and other required FDA documentation as outlined in Section 312.32 IND safety reports, revised April 1, 2010.

Safety Evaluations:

Risks for Study Personnel: There is minimal risk involved to study personnel. A trained laboratory phlebotomist will draw patient blood for laboratory analysis and serum/blood storage. There is a slight risk of potential needle stick, however the Durham VA Chemical Laboratory provides extensive risk prevention training for all phlebotomists, thus the likelihood of an accidental needle stick is low. The blood samples will be transported to -80° freezer for storage by study personnel. In order to reduce the risk of direct exposure, personnel will wear personal protective equipment while transporting patient serum/blood.

Medical Monitoring and Physician On-Call Schedule / Cascade:

Dr. Christine Marx will be the 1st-call physician for any medical issues involving the proposed randomized controlled trial with an adjunctive neurosteroid intervention. If Dr. Marx is not immediately accessible within 5 minutes on her cell phone, then Dr. Steven Szabo will be called on his cell phone - who will function as the 2nd-line physician if Dr. Marx is unavailable. Dr. Szabo completed his residency training at Duke University Medical Center and has extensive psychiatric research experience. Should Dr. Szabo not be available, the cell phone cascade would then move to the 3rd-line physician, Dr. Dan Bradford. Dr. Bradford has extensive experience in the conduction of randomized controlled trials, and has collaborated with Dr. Marx for several years. Dr. Mike Hertzberg would provide 4th line coverage. Dr. Hertzberg also has extensive experience in the conduction of randomized controlled trials enrolling Veteran cohorts; Dr. Hertzberg and Dr. Marx frequently cover for each other's clinical trials if one of them is traveling or on annual leave.

There will be a master schedule available that adapts the above call-schedule to the annual leave and travel schedules of the individual MDs listed in this cascade. This on-call schedule will be circulated weekly, so that the order of the call cascade is always up-to-date for any particular week (and incorporates any changes that may be necessary secondary to individual MD schedules).

The designated physician on-call will have the following responsibilities: The designated study team MD will conduct physical exams at baseline and study completion, medical chart review, determination of patient eligibility for study entry, review of all clinical laboratory results and ECG tracings within 24 hours (with ECG confirmation by cardiologist Joseph Greenfield, MD), assessments of potential side effects to study medication by reviewing a structured rating assessment administered to each research participant at each study visit and by interviewing and examining the participant as clinically indicated, and will attend to any other medical issues that arise during the course of this study.

Should any emergent issues arise that require immediate medical and/or psychiatric attention, patients will be walked to the Durham VA Emergency Department and Psychiatric Emergency Care (PEC) services –

which provide acute medical and psychiatric care 24 hours per day/7 days per week. The PEC is staffed by a psychiatry resident, psychiatric social worker, and a VA psychiatry attending.

A clinical warning indicating participation in this research study will be added to the participant's CPRS medical record. The clinical warning will be removed when the participant completes study involvement.

PAYMENTS TO SUBJECTS

Subjects will be paid $75.00 for each study visit (six study visits total, or $450.00 total if a subject completes all six study visits). If a subject attends two study visits, the subject will be paid $150; if s/he attends 3 study visits, will be paid $225, etc.). There are no research-related costs to subjects participating in this study. Participants will be paid by direct deposit or provided with a paper voucher, which is redeemable for cash at the Durham VAMC Cashier's Office (payment for funds will be provided by the VA, as this study is funded by a VA Career Development Award).

There will be an additional allowance for travel to the Durham VAMC based on the distance traveled by the subject. The travel allowance will be approximated by the distance traveled. Travel allowance for several cities and major towns in VISN6 are included in the table below. There will be no further compensation for meals, parking, childcare, lost wages, etc.

Distance (roundtrip miles)	Allowance	Typical towns and cities
0-50	$10	Raleigh, Durham, Cary, Chapel Hill
50-100	$20	Henderson, Wake Forest, Burlington
100-200	$30	Greensboro, Fayetteville, Goldsboro
200-300	$40	Salisbury, Greenville, Rocky Mount
300-400	$50	Wilmington, Charlotte, Hickory
> 400	$60	

DATA AND SAFETY MONITORING

Sources of Materials

All of the data for this study will be collected specifically for research purposes. All study data will be kept in locked file cabinets, in locked offices within locked (card accessible only) buildings and will be accessible only to qualified research personnel [REDACTED]. All locations are research controlled space). Each subject will be assigned a study number and all data and specimens will be identified by that study number. The list linking this number to subject identity will be kept in a file on a secure VA network drive [REDACTED], accessed through a password-protected computer to which only the Principal Investigator and study staff have access. Five sources of data will be maintained for this study and all collected information will be coded: a) the initial telephone screening, b) diagnostic interviews, c) physical examinations, d) psychiatric rating scales (both self-report and clinician-administered, e) neurosteroid profiles and other laboratory measurements.

Data Monitoring and Confidentiality

Reports from subjects' clinical records concerning research observations will not be made available to outside medical facilities without the written consent of the patient. All clinical and biological data obtained from research interviews and the laboratory will be coded. The coded data will be kept in locked file cabinets, in locked offices within locked (card accessible only) buildings and will be accessible only to qualified research personnel [REDACTED]. Only study numbers will appear on data, documents and biological specimens used for evaluation or statistical analysis (ie, coded). The key-code linking subjects to study data identified by study

number will be kept on a secure VA network drive [REDACTED]. In addition, any publications resulting from this research will not identify individual subjects.

Only members of our DVAMC research team will have access to identifiers, whether they reside behind the VA firewall or in paper form. Coded data will be sent via the Safe Access File Exchange (SAFE) at https://safe.amrdec.army.mil/safe/ to Duke University for statistical computing purposes (including Ryan Wagner, PhD). SAFE is a secure method of exchanging files <2GB to and from individuals with a valid .gov, .mil, .com, or .edu email addresses.

PRIVACY AND CONFIDENTIALITY

Patient confidentiality will be maintained through the assignment of patient identification numbers. These numbers will be used in keeping of all research records. All hard copy research materials will be kept in locked file cabinets with keys available only to the principal investigator, and research personnel participating directly in this protocol. Patients will be informed during the consent process about the limits of confidentiality.

Information about study subjects will be kept confidential and managed according to the requirements of the Health Insurance Portability and Accountability Act of 1996 (HIPAA). Those regulations require a signed subject authorization informing the subject of the following:

-What protected health information (PHI) will be collected from subjects in this study
-Who will have access to that information and why
-Who will use or disclose that information
-The rights of a research subject to revoke their authorization for use of their PHI.

PHI collected as part of this study will include: name, address, telephone number, date of birth, dates of service, dates of attendance, social security number, gender, close relative's name, close relative phone number, marital status, race, education level, work status, employer, length of employment, occupation, service connection (reason, percentage), disability status, medical and mental health diagnoses and treatments, medication, substance use, history of suicide attempts, family history of suicide attempts, smoking status, incarceration history, twin status, military service (time period, branch, number of tours, whether or not subjects served in a war zone, fired a weapon in a combat situation, were under enemy fire, were wounded in a war zone, awarded medals or were prisoners of war). Genetic data and study visit and assessment dates will also be collected.

As part of the study (and as indicated in the HIPAA Authorization), patient information and medical and/or research records may be disclosed to the Office for Human Research Protections (OHRP), the VA Office of the Inspector General (OIG), the Office of Research Oversight (ORO), other government agencies, the Durham VAMC Institutional Review Board (IRB), and/or local Research Compliance Officers, and Food and Drug Administration (FDA).

In the event that a subject revokes authorization to collect or use PHI, the investigator, by regulation, retains the ability to use all information collected prior to the revocation of subject authorization. For subjects that have revoked authorization to collect or use PHI, attempts will be made to obtain permission to collect at least vital status (i.e., that the subject is alive) at the end of their scheduled study period.

INFORMATION SECURITY

Collection, Use and Storage of Research Information

Participant data will be stripped of HIPAA identifiers listed in Appendix B of the VHA Handbook 1605.1 (except for study date), and participant information will be identifiable only by a unique, linked numerical code (i.e., coded data). The master key code will be maintained on the secure VA network drive [REDACTED] and which is accessible only through a VA, password protected computer [REDACTED]. All electronically recorded PHI (i.e., any patient identifying information) will be maintained on a secure VA password protected network drive [REDACTED] on a VA computer. Any PHI collected on paper which needs to be maintained (ie, Informed Consent Form, HIPAA Authorization, Subject Payment Forms, etc) will be stored in a folder separate from coded paper forms (i.e., study assessments). These two folders will be filed in two different locked file cabinets inside a locked room within the Durham VAMC [REDACTED]. Only approved VA researchers and study personnel will have access to this information. No PHI will be removed from the Durham VAMC environment. Coded information collected using paper forms (i.e., study assessments) will be stored in a locked filing cabinet [REDACTED]. These paper forms will be entered into a database stored on a separate, secure, password protected database [REDACTED] within the Durham VAMC (so that information cannot be linked to participant PHI) by study personnel and will be stored indefinitely according to VA guidelines. Paper form and electronic data will be destroyed in accordance with the Records Control Schedule.

Patient blood and serum samples will be collected during the study visit(s) and coded with a corresponding unique, linked numerical code (ie, the study ID number). Coded blood and serum samples will be stored at the Durham VAMC in a locked freezer room in [REDACTED] located in secure research wings (requires special permission and a security activated badge to gain access) within the Durham VAMC. At different points, a small portion of this blood will be hand-carried by study personnel to Duke University for the purposes of pharmacogenetic and genetic analysis only. Any remaining samples will be returned to the Durham VA after analyses have been completed. Data derived from blood and serum samples will be returned to the VA thru the SAFE procedure.

Subjects will be asked to provide permission to store their contact and demographic information and study interview results in a secure database (an excel spreadsheet maintained by the PI and her study staff on a secure VA server) to be used to select potentially eligible participants for other ongoing and future studies. If patients provide permission to do so, they may then be re-contacted in the future and invited to participate in other studies for which they may qualify, and of which Drs. Naylor and/or Marx are investigators. Demographic information asked will include: name, social security number, birthdate, gender, address, telephone number, close relative's name, close relative phone number, marital status, race, education level, work status, employer, length of employment, occupation, service connection (reason, percentage), disability status, history of hospitalization for schizophrenia, schizoaffective disorder or PTSD—if yes, age of diagnosis, family history of schizophrenia or schizoaffective disorder, history of suicide attempts, family history of suicide attempts, smoking status—if yes, age of start, or quit, incarceration history, twin status, military service (time period, branch, number of tours, whether or not subjects served in a war zone, fired a weapon in a combat situation, were under enemy fire, were wounded in a war zone, awarded medals or were prisoners of war).

Any incident regarding theft/loss of data, authorized access of sensitive data or storage devices or non-compliance with security controls will be addressed according to VA policy (VHA Handbook 1200.05, 10j; 1058.01 11.a; 6500, Appendix D and 6500.2) and will be reported to Durham VAMC PO and ISO immediately using the following: VHADURResearchEventReport@va.gov.

Study members who are initially part of the study team, but leave the study team for whatever reason will no longer have access to participant study data.

DATA ANALYSIS AND STATISTICAL CONSIDERATIONS

Statistical Methods

Analysis of treatment effects will use the mixed-models approach to repeated-measures analysis of variance (SAS Institute, Cary, NC) with baseline and endpoint scores as dependent variables, time as a within-subject repeated measure, and treatment group (pregnenolone or placebo) as a between-subjects fixed factor. The primary analyses for this will be based on an intention-to-treat principle that will include in the analyses all randomized subjects. The primary efficacy outcome variable is the weekly mean of the average pain scores (0 – 10 numeric rating pain score recorded daily from patient) after study drug initiation. These are obtained over time, and thus we will use mixed effects models with random intercepts to account for subject effects and the correlation among repeated measurements within a subject. Models will be developed which include as independent variables the treatment group and the baseline pain score. Thus, the effect of the intervention will be assessed by examining the significance of the slope β_1 coefficient for intervention in the following model:

$$P_{it} = \beta_0 + b_{0i} + \beta_1 Int + \beta_2 BaseP + \beta_3 t + e_{it}$$

where P_{it} is the mean weekly pain score for the ith individual at time point t, β's denote fixed effects, b_{0i} is the random intercept for the ith individual, and e's are random errors. Patients who are withdrawn from the study because of side effects will only be included in outcome assessments in which they participated. The mixed effects models can handle incomplete panels of information. All tested hypotheses will be considered statistically significant if the 2 sided p value is ≥0.05 unless otherwise specified.

Secondary analyses will include the following assessments. Assessment of changes in mood, anxiety, sleep, working memory, executive function, and pain interference (BDI, DTS, Tower of London, Digit Sequencing Task, BPI interference, PGI, AIS) will be analyzed via a repeated measures analysis similar to analyses for primary outcome assessments. Categorical analyses of patient response (30% improvement, 50% improvement) will be evaluated via a comparison of proportions using Fisher's exact test. Time to event analyses will also be performed, to examine the time to 30% and time to 50% reduction in 24 hour average pain score, using daily pain score data. These analyses will be performed by calculating Kaplan-Meier survival curves. Changes in physical function, mental health, cognitive symptoms and back pain-related disability will be assessed using ANCOVA models Safety will be assessed by comparing the incidence of major and minor side effects (defined in human subjects section below) in the study drug vs. placebo groups, using Fisher's exact tests. Path analysis will be used to perform a preliminary assessment of whether any pain reduction effect is independent of possible effects of study drug on anxiety and mood.

Missing data: The mixed models used to evaluate the continuous response variables are able to handle moderate amounts of missing data provided they are missing at random. We will examine the missing at random assumption by assessing differences between dropouts and completers on baseline as well as response variables up to point of premature withdrawal. If the missing at random assumption does not appear to be tenable, we will report the mixed models results but spend additional effort characterizing treatment effect at time of premature withdrawal.

Power Analysis and Sample Size Determination:

The effect of pregnenolone on low back pain will be evaluated using several statistical methods. This clinical trial design will compare two randomly assigned groups (pregnenolone or placebo) with respect to self-reported lower back pain measured on a 0-10 pain scale. Inclusion criteria for the study require that all participants report pain severity of at least 4/10. Given the low back pain often presents as long periods of low-level intensity pain punctuated by periods of acute exacerbation, it is quite likely that the mean pain level for

selected participants will substantially exceed 4/10. Thus for power calculations, we assume a baseline mean pain level of six for each group. We also assume a standard deviation of 1.0 since we have little information to support any specific value in this population.

For the purpose of power calculations we assume that the mean pain levels of the two independent groups will be compared using a Two-Sample T-Test**,** Null Hypothesis: Mean1=Mean2. Alternative Hypothesis: Mean1<>Mean2. The standard deviations were assumed to be unknown and equal.

Assuming specified numbers of participants in each group (N) the mean differences detectable for at least 80% power and alpha=0.05 are shown in the following table and figure.

**N1**	**Mean1**	**Mean2**
4	6.0	3.6
5	6.0	3.8
6	6.0	4.0
6	6.0	4.2
8	6.0	4.4
10	6.0	4.6
12	6.0	4.8
17	6.0	5.0
26	6.0	5.2
45	6.0	5.4
100	6.0	5.6

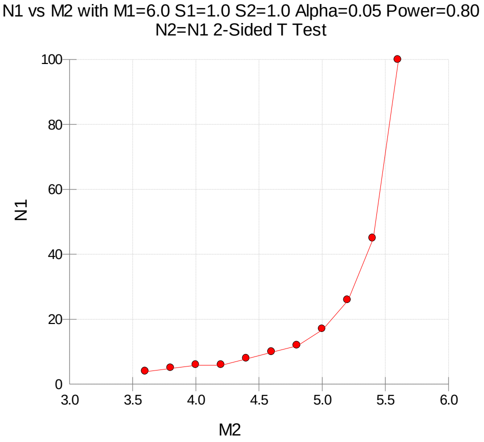

Group sample sizes of 45 achieve at least 80% power to detect a difference of 0.6 between the null hypothesis that both group means are 6.0 and the alternative hypothesis that the mean of group 2 is 5.4 with estimated group standard deviations of 1.0 and 1.0 and with a significance level (alpha) of 0.05000 using a two-sided two-sample t-test. Using this experimental group size should yield sufficient power to detect clinical meaningful effects.

Data Collection: Data pertaining to psychiatric rating and other assessment scales will be collected on hard copy and data pertaining to laboratory values will be collected in manners used for the VA Mid-Atlantic MIRECC Registry. A study number identifying clinical and biological data will be the only number to appear on data and documents used for evaluation or statistical analysis. This data will be kept in locked file cabinets accessible only to qualified personnel.

REFERENCES

Belelli D., Bolger M.B., Gee K.W. (1989) Anticonvulsant profile of the progesterone metabolite 5 alphapregnan-3 alpha-ol-20-one. Eur J Pharmacol 166:325-9.

Bilmes, L. (2007). Solders returnign from Iraq and Afghanistan: The long-term costs of providing veterans medical care and disabiilty benefits. John F. Kennedy School of Government--Harvard University, RWP07-001.

Bitran D., Klibansky D.A., Martin G.A. (2000) The neurosteroid pregnanolone prevents the anxiogenic-like effect of inescapable shock in the rat. Psychopharmacology (Berl) 151:31-7.

Cherkin, D.C., Sherman, K.J., Avins, A.L., Erro, J.H., Ichikawa, L., Barlow, W.E., Delaney, K., Hawkes, R., Hamilton, L., Pressman, A., Khalsa, P.S., Devo, R.A. (2009) A randomized trial comparing acupuncture, simulated acupuncture, and usual care for chronic low back pain. Arch Intern Med. May 11; 169(9): 858-66.

Clark M.E., Bair M.J., Buckenmaier C.C., 3rd, Gironda R.J., Walker R.L. (2007) Pain and combat injuries insoldiers returning from Operations Enduring Freedom and Iraqi Freedom: implications for research and practice. J Rehabil Res Dev 44:179-94.

Crawley J.N., Glowa J.R., Majewska M.D., Paul S.M. (1986) Anxiolytic activity of an endogenous adrenal steroid. Brain Res 398:382-5.

Cutler S.M., Cekic M., Miller D.M., Wali B., VanLandingham J.W., Stein D.G. (2007) Progesterone improvesacute recovery after traumatic brain injury in the aged rat. J Neurotrauma 24:1475-86.

Devaud L.L., Purdy R.H., Morrow A.L. (1995) The neurosteroid, 3 alpha-hydroxy-5 alpha-pregnan-20-one, protects against bicuculline-induced seizures during ethanol withdrawal in rats. Alcohol Clin Exp Res 19:350-5.

Djebaili M., Hoffman S.W., Stein D.G. (2004) Allopregnanolone and progesterone decrease cell death andcognitive deficits after a contusion of the rat pre-frontal cortex. Neuroscience 123:349-59.

Djebaili M., Guo Q., Pettus E.H., Hoffman S.W., Stein D.G. (2005) The neurosteroids progesterone and allopregnanolone reduce cell death, gliosis, and functional deficits after traumatic brain injury in rats. J Neurotrauma 22:106-18.

Ebert, M., Kerns, R.D. (2011). Behavioral and Psychopharmacologic Pain Management: Psychopharmacologic, Behavioral and Psychtherapeutic Approaches. Cambridge University Press. Pg 114-270.

Frye C.A., Duncan J.E. (1994) Progesterone metabolites, effective at the GABAA receptor complex, attenuate pain sensitivity in rats. Brain Res 643:194-203.

Gatchel, R.J., Peters, M.L., Fuchs, P.N., Turk, D.C. (2007) The Biopsychosocial Approach to Chronic Pain:Scientific Advances and Future Directions. Psychological Bulletin. Vol 133, No 4, 581-624.

Gawande A. (2004) Casualties of war--military care for the wounded from Iraq and Afghanistan. N Engl J Med 351:2471-5.

Ghoumari A.M., Ibanez C., El-Etr M., Leclerc P., Eychenne B., O'Malley B.W., Baulieu E.E., Schumacher M. (2003) Progesterone and its metabolites increase myelin basic protein expression in organotypic slicecultures of rat cerebellum. J Neurochem 86:848-59.

Gironda R.J., Clark M.E., Ruff R.L., Chait S., Craine M., Walker R., Scholten J. (2009) Traumatic brain injury,polytrauma, and pain: challenges and treatment strategies for the polytrauma rehabilitation. Rehabil Psychol 54:247-58.

Guidotti A., Dong E., Matsumoto K., Pinna G., Rasmusson A.M., Costa E. (2001) The socially-isolated mouse: a model to study the putative role of allopregnanolone and 5alpha-dihydroprogesterone in psychiatric disorders. Brain Res Brain Res Rev 37:110-5. DOI: S0165017301001291 [pii].

He J., Evans C.O., Hoffman S.W., Oyesiku N.M., Stein D.G. (2004) Progesterone and allopregnanolone reduce inflammatory cytokines after traumatic brain injury. Exp Neurol 189:404-12.

Helmer, D. A., H. K. Chandler, et al. (2009). Chronic widespread pain, mental health, and physical role functionin OEF/OIF veterans. Pain Med 10(7): 1174-1182.

Hoge C.W., Castro C.A., Messer S.C., McGurk D., Cotting D.I., Koffman R.L. (2004) Combat duty in Iraq and Afghanistan, mental health problems, and barriers to care. N Engl J Med 351:13-22.

Hoge C.W., McGurk D., Thomas J.L., Cox A.L., Engel C.C., Castro C.A. (2008) Mild traumatic brain injury inU.S. Soldiers returning from Iraq. N Engl J Med 358:453-63.

Institute of Medicine of the National Academies Report (2011). Relieving Pain in America: A Blueprint for Transforming Prevention, Care, Education, and Research. The National Academies Press,Washington DC.

Jain N.S., Hirani K., Chopde C.T. (2005) Reversal of caffeine-induced anxiety by neurosteroid 3-alphahydroxy-5-alpha-pregnane-20-one in rats. Neuropharmacology 48:627-38.

Kavaliers M. (1988) Inhibitory influences of the adrenal steroid, 3 alpha, 5 alpha-tetrahydrodeoxycorticosterone[correction of tetrahydroxycorticosterone] on aggression and defeat-induced analgesia in mice. Psychopharmacology (Berl) 95:488-92.

Kavaliers M., Wiebe J.P. (1987) Analgesic effects of the progesterone metabolite, 3 alpha-hydroxy-5 alphapregnan-20-one, and possible modes of action in mice. Brain Res 415:393-8.

Kavaliers M., Perrot-Sinal T.S., Desjardins D.C., Cross-Mellor S.K., Wiebe J.P. (2000) Antinociceptive effects of the neuroactive steroid, 3alpha-hydroxy-5alpha-pregnan-20-one and progesterone in the land snail, Cepaea nemoralis. Neuroscience 95:807-12.

Keefe, F.J., Shelby, R.S., Somers, T.J., Varia, I., Blazing, M., Waters, S.J., McKee, D., Silva S., She, L., Blumenthal, J.A., O-Connor, J., Knowles, V., Johnson, P., Bradley, L. (2011). Effects of coping skills training and sertraline in patients with non-cardiac chest ain: a randomized controlled study. Pain. Apr; 152 (4):730-41.

Kerns R.D., Otis J., Rosenberg R., Reid M.C. (2003) Veterans' reports of pain and associations with ratings ofhealth, health-risk behaviors, affective distress, and use of the healthcare system. J Rehabil Res Dev 40:371-9.

Kerns, R.D., Sellinger, J., Goodin, B.R. (2011) Psychological Treatment of Chronic Pain. Annu Rev. Clin Psychol. 7:411-34

Kilts J.D., Tupler L.A., Keefe F.J., Payne V.M., Hamer R.M., Naylor J.C., Calnaido R.P., Morey R.A., Strauss J.L., Parke G., Massing M.W., Youssef N.A., Shampine L.J., Marx C.E. (2010) Neurosteroids and self reportedpain in veterans who served in the U.S. Military after September 11, 2001. Pain Med 11:1469-76.

Kokate T.G., Svensson B.E., Rogawski M.A. (1994) Anticonvulsant activity of neurosteroids: correlation with gamma-aminobutyric acid-evoked chloride current potentiation. J Pharmacol Exp Ther 270:1223-9.

Kokate T.G., Cohen A.L., Karp E., Rogawski M.A. (1996) Neuroactive steroids protect against pilocarpine- and kainic acid-induced limbic seizures and status epilepticus in mice. Neuropharmacology 35:1049-56.

Korneyev A., Costa E. (1996) Allopregnanolone (THP) mediates anesthetic effects of progesterone in rat brain.Horm Behav 30:37-43.

Landgren S., Aasly J., Backstrom T., Dubrovsky B., Danielsson E. (1987) The effect of progesterone and its metabolites on the interictal epileptiform discharge in the cat's cerebral cortex. Acta Physiol Scand 131:33-42.

Lew H.L., Otis J.D., Tun C., Kerns R.D., Clark M.E., Cifu D.X. (2009) Prevalence of chronic pain, posttraumatic stress disorder, and persistent postconcussive symptoms in OIF/OEF veterans: polytrauma clinical triad. J Rehabil Res Dev 46:697-702.

Majewska M.D., Harrison N.L., Schwartz R.D., Barker J.L., Paul S.M. (1986) Steroid hormone metabolites are barbiturate-like modulators of the GABA receptor. Science 232:1004-7.

Marx C.E., Kilts J.D., Naylor J.C., Shampine L.J., Strauss J.L., VA-Mid-Atlantic-MIRECC-Workgroup, TuplerL.A. (2010) Neurosteroids are Altered in Veterans who Sustained a Blast-Related Traumatic Brain Injury (TBI) in Iraq or Afghanistan., American College of Neuropsychopharmacology Annual Meeting, Miami, FL.

Marx C.E., Shampine L.J., Khisti R.T., Trost W.T., Bradford D.W., Grobin A.C., Massing M.W., Madison R.D., Butterfield M.I., Lieberman J.A., Morrow A.L. (2006) Olanzapine and fluoxetine administration andcoadministration increase rat hippocampal pregnenolone, allopregnanolone and peripheral deoxycorticosterone: implications for therapeutic actions. Pharmacol Biochem Behav 84:609-17.

Mechlin B., Morrow A.L., Maixner W., Girdler S.S. (2007) The relationship of allopregnanolone immunoreactivity and HPA-axis measures to experimental pain sensitivity: Evidence for ethnic differences. Pain 131:142-52.

Mok W.M., Krieger N.R. (1990) Evidence that 5 alpha-pregnan-3 alpha-ol-20-one is the metabolite responsible for progesterone anesthesia. Brain Res 533:42-5.

Morrow A.L., Suzdak P.D., Paul S.M. (1987) Steroid hormone metabolites potentiate GABA receptor-mediated chloride ion flux with nanomolar potency. Eur J Pharmacol 142:483-5.

Nampiaparampil D.E., Nampiaparampil J.X., Harden R.N. (2009) Pain and prejudice. Pain Med 10:716-21.

Naylor, JC, Payne, VM, Youssef, N, Strauss, JL, Kilts, JD, Capehart, B, Keefe, R, Davidson, JRT., Marx, CE. Pilot Study of Aripiprazole as a Therapeutic Strategy in OEF/OIF-Era Veterans with PTSD. Poster presented at the International Society for Traumatic Stress Studies, November 2010, Montreal, Canada.

Pathirathna S., Brimelow B.C., Jagodic M.M., Krishnan K., Jiang X., Zorumski C.F., Mennerick S., Covey D.F., Todorovic S.M., Jevtovic-Todorovic V. (2005) New evidence that both T-type calcium channels and GABAA channels are responsible for the potent peripheral analgesic effects of 5alpha-reduced neuroactive steroids. Pain 114:429-43.

Payne V., Morey R., Tupler L., Keefe F., Marx C. (2007) Neuroactive Steroids and Self-Reported Pain in Veterans who Served During OEF/OIF, 23rd International Society for Traumatic Stress Studies Annual Meeting, Baltimore, MD. Pettus E.H., Wright D.W., Stein D.G., Hoffman S.W. (2005) Progesterone treatment inhibits the inflammatory agents that accompany traumatic brain injury. Brain Res 1049:112-9.

Pinna G., Dong E., Matsumoto K., Costa E., Guidotti A. (2003) In socially isolated mice, the reversal of brain allopregnanolone down-regulation mediates the anti-aggressive action of fluoxetine. Proc Natl Acad Sci U S A 100:2035-40.

Rasmusson A.M., Pinna G., Paliwal P., Weisman D., Gottschalk C., Charney D., Krystal J., Guidotti A. (2006) Decreased cerebrospinal fluid allopregnanolone levels in women with posttraumatic stress disorder. Biol Psychiatry 60:704-13.

Roof R.L., Hoffman S.W., Stein D.G. (1997) Progesterone protects against lipid peroxidation following traumatic brain injury in rats. Mol Chem Neuropathol 31:1-11.

Samwell, H.J., Krassimaat, F.W., Crul, B.J., van Dongen, R.D. , Evers A.W. (2009) Multidisciplinary allocation of chronic pain treatment: effects and cognitive-behavioural predictors of outcome. Br J Health Psychol. Sept 14 (Pt 3): 405-21.

Sayeed I., Guo Q., Hoffman S.W., Stein D.G. (2006) Allopregnanolone, a progesterone metabolite, is more effective than progesterone in reducing cortical infarct volume after transient middle cerebral artery occlusion. Ann Emerg Med 47:381-9.

Sayeed I., Parvez S., Wali B., Siemen D., Stein D.G. (2009) Direct inhibition of the mitochondrial permeability transition pore: a possible mechanism for better neuroprotective effects of allopregnanolone over progesterone. Brain Res 1263:165-73.

Schneiderman A.I., Braver E.R., Kang H.K. (2008) Understanding sequelae of injury mechanisms and mild traumatic brain injury incurred during the conflicts in Iraq and Afghanistan: persistent postconcussive symptoms and posttraumatic stress disorder. Am J Epidemiol 167:1446-52.

Schule C., Romeo E., Uzunov D.P., Eser D., di Michele F., Baghai T.C., Pasini A., Schwarz M., Kempter H., Rupprecht R. (2006) Influence of mirtazapine on plasma concentrations of neuroactive steroids in major depression and on 3alpha-hydroxysteroid dehydrogenase activity. Mol Psychiatry 11:261-72.

Shipherd J.C., Keyes M., Jovanovic T., Ready D.J., Baltzell D., Worley V., Gordon-Brown V., Hayslett C., Duncan E. (2007) Veterans seeking treatment for posttraumatic stress disorder: what about comorbid chronic pain? J Rehabil Res Dev 44:153-66.

Stein D.G. (2008) Progesterone exerts neuroprotective effects after brain injury. Brain Res Rev 57:386-97.

Stein D.G., Wright D.W., Kellermann A.L. (2008) Does progesterone have neuroprotective properties? Ann Emerg Med 51:164-72.

Strohle A., Romeo E., Hermann B., Pasini A., Spalletta G., di Michele F., Holsboer F., Rupprecht R. (1999) Concentrations of 3 alpha-reduced neuroactive steroids and their precursors in plasma of patients with major depression and after clinical recovery. Biol Psychiatry 45:274-7.

Tanielian T., Jaycox L.H., Schell T.L., Marshall G.N., Burnam M.A., Eibner C., Karney B.R., Meredith L.S.,Ringel J.S., Vaiana M.E., Team a.t.I.W.S. (2008) Invisible Wounds of War, Rand Corporation. pp. 1-66.

Taylor, B. C., E. M. Hagel, et al. (2012). Prevalence and costs of co-occurring traumatic brain injury with and without psychiatric disturbance and pain among Afghanistan and Iraq War Veteran V.A. users. Med Care 50(4): 342-346.

Toomey R., Kang H.K., Karlinsky J., Baker D.G., Vasterling J.J., Alpern R., Reda D.J., Henderson W.G., Murphy F.M., Eisen S.A. (2007) Mental health of US Gulf War veterans 10 years after the war. Br J Psychiatry 190:385-93.

Trigkilidas, D., (2010). Acupuncture therapy for chronic lower back pain: a systemic review. Ann R Coll Surg Engl. Oct; 92 (7): 595-8.

Uzunova V., Sheline Y., Davis J.M., Rasmusson A., Uzunov D.P., Costa E., Guidotti A. (1998) Increase in the cerebrospinal fluid content of neurosteroids in patients with unipolar major depression who are receiving fluoxetine or fluvoxamine. Proc Natl Acad Sci U S A 95:3239-44.

VanLandingham J.W., Cekic M., Cutler S., Hoffman S.W., Stein D.G. (2007) Neurosteroids reduce inflammation after TBI through CD55 induction. Neurosci Lett 425:94-8.

Walker W.C., Seel R.T., Curtiss G., Warden D.L. (2005) Headache after moderate and severe traumatic brain injury: a longitudinal analysis. Arch Phys Med Rehabil 86:1793-800.

Warden D.L., French L. (2005) Traumatic brain injury in the war zone. N Engl J Med 353:633-4.

Wieland S., Lan N.C., Mirasedeghi S., Gee K.W. (1991) Anxiolytic activity of the progesterone metabolite 5 alpha-pregnan-3 alpha-o1-20-one. Brain Res 565:263-8.

Winfree C.J., Coombs D.W., DeLeo J.A., Colburn R.W. (1992) Analgesic effects of intrathecally-administered 3 alpha-hydroxy-5 alpha-pregnan-20-one in a rat mechanical visceral pain model. Life Sci 50:1007-12.

Wolkowitz O.M., Reus V.I., Keebler A., Nelson N., Friedland M., Brizendine L., Roberts E. (1999) Double-blind treatment of major depression with dehydroepiandrosterone. Am J Psychiatry 156:646-9.

Wright D.W., Kellermann A.L., Hertzberg V.S., Clark P.L., Frankel M., Goldstein F.C., Salomone J.P., Dent L.L., Harris O.A., Ander D.S., Lowery D.W., Patel M.M., Denson D.D., Gordon A.B., Wald M.M., Gupta S., Hoffman S.W., Stein D.G. (2007) ProTECT: a randomized clinical trial of progesterone for acute traumatic brain injury. Ann Emerg Med 49:391-402, 402 e1-2.

Xiao G., Wei J., Yan W., Wang W., Lu Z. (2008) Improved outcomes from the administration of progesterone for patients with acute severe traumatic brain injury: a randomized controlled trial. Crit Care 12:R61.

Young A.H., Gallagher P., Porter R.J. (2002) Elevation of the cortisol-dehydroepiandrosterone ratio in drugfree depressed patients. American Journal of Psychiatry 159:1237-9.

Youssef NA N.J., Massing M,Kilts J,Strauss J, Keefe R, Shampine L, Allen T, Marx CE. (2010) Pregnenolonefor Cognitive and PTSD Symptoms in Veterans with Mild TBI: A proof-of-concept Randomized Controlled Trial., VA Mental Health Meeting Baltimore, MD.

VHA Office of Public Health and Environmental Hazards. Analysis of VA health care utilization among operation enduring freedom (OEF) and operation Iraqi freedom (OIF) veterans. Retrieved from VHA Office of Public Health and Environmental Hazards Web (2010a).

Pain Management Task Force:1-163 (2010b).

Management of Opioid Therapy for Chronic Pain Working Group. VA/DoD clinical practice guideline for management of opioid therapy for chronic pain. Department of Veterans Affairs, Department of Defense:159 (2010c).
