# Supplementary material for: Effect of Pregnenolone vs Placebo on Self-reported Chronic Low Back Pain Among US Military Veterans: A Randomized Clinical Trial
Source: JAMA Netw Open. 2020 Mar 2;3(3):e200287. doi: 10.1001/jamanetworkopen.2020.0287 (PMC7052727; doi:10.1001/jamanetworkopen.2020.0287)
Supplement: Supplement 2. — eTable 1. Adverse Events as Reported on Hillside Adverse Event Scale eTable 2. Odds Ratios for 20% Improvement of Pain Based on Treatment with Pregnenolone vs Placebo eTable 3. Secondary Outcomes eTable 4. Neurosteroid Level Changes by Treatment Group [file jamanetwopen-3-e200287-s002.pdf]

Supplementary Online Content

Naylor JC, Kilts JD, Shampine LJ, et al. Effect of pregnenolone vs placebo on self-reported chronic low back pain among US military veterans: a randomized clinical trial. *JAMA Netw Open*. 2020;3(3):e200287. doi:10.1001/jamanetworkopen.2020.0287

eTable 1. Adverse Events as Reported on Hillside Adverse Event Scale
eTable 2. Odds Ratios for 20% Improvement of Pain Based on Treatment with Pregnenolone vs Placebo
eTable 3. Secondary Outcomes
eTable 4. Neurosteroid Level Changes by Treatment Group

This supplementary material has been provided by the authors to give readers additional information about their work.

© 2020 Naylor JC et al. *JAMA Network Open.*

eTable 1. Adverse Events as Reported on Hillside Adverse Event Scale

	**Placebo**		**Pregnenolone**	
	Total Number of Participants Reporting Symptoms	Total Times Reported on Hillside	Total Number of Participants Reporting Symptoms	Total Times Reported on Hillside
**Symptom**				
Delirium/Confusion	0	0	2	3
Disorientation	1	1	2	2
Excitement/Agitation	0	0	5	5
Restlessness	1	2	2	2
Increased motor activity	1	1	2	2
Decreased motor activity	2	2	0	0
Malaise	0	0	2	3
Insomnia	8	11	6	8
Hypersomnia	2	2	1	1
Drowsiness	7	7	8	9
Myoclonus	1	1	2	2
Cramps	2	2	5	5
Tremor	1	2	2	2
Paresthesia	1	1	4	4
Dyskinesia	0	0	2	2
Tinnitus	5	5	6	6
Vertigo	1	1	2	3
Hypertension	1	1	2	2
Syncope/Dizziness	2	4	2	2
Palpitations	1	1	1	1
Tachycardia	0	0	1	1
Peripheral edema	0	0	1	1
Cold extremities	1	1	1	1
Dry mouth	8	8	5	7
Nasal congestion	2	2	4	4
Blurred vision	1	1	1	1
Constipation	2	2	3	4
Increased salivation	2	2	0	0
Fever	1	1	1	1
Sweating	0	0	1	1
Nausea	4	4	7	8
Vomiting	2	2	2	2
Diarrhea	3	3	6	8
Urinary retention	2	2	0	0
Nocturnal/Enuresis	0	0	1	1
Menstrual disturbance	1	1	2	2
Decreased interest in sex	1	2	2	2
Impaired sex performance	0	0	2	4
Dermatological	5	6	3	2
Joint pain/stiffness	4	7	4	5
Muscle pain/stiffness	7	9	7	7
Decreased appetite	4	5	7	8

© 2020 Naylor JC et al. *JAMA Network Open.*

eTable 2. Odds Ratios for 20% Improvement of Pain based on Treatment with Pregnenolone vs Placebo

	**Pain Diary 20%**				**Pain Recall 20%**		
	**No**	**Yes**	**Total**		**No**	**Yes**	**Total**
**Placebo**	30	12	42	**Placebo**	31	11	42
	71.4%	28.6%			73.8%	26.2%	
**Pregnenolone**	20	21	41	**Pregnenolone**	21	20	41
	48.8%	51.2%			51.2%	48.8%	
**Total**	50	33	83	**Total**	52	31	83
**OR**	**-95%CI**	**+95%CI**	**p-Value**	**OR**	**-95%CI**	**+95%CI**	**p-Value**
2.62	1.06	6.50	**0.04**	2.68	1.07	6.74	**0.04**

© 2020 Naylor JC et al. *JAMA Network Open.*

eTable 3 Secondary Outcomes

Secondary Outcome at Visit 6	Est (SE)	z-Value	p-Value
**Sleep (AIS)**			
Placebo	6.88 (0.46)		
Pregnenolone	6.29 (0.39)		
Difference	0.59 (0.92)	-1.07	0.29
**Resilience (CD-RISC)**			
Placebo	73.13 (0.94)		
Pregnenolone	73.21 (1.43)		
Difference	-0.08 (1.54)	0.05	0.96
**Working Memory (DS)**			
Placebo	22.74 (0.34)		
Pregnenolone	22.26 (0.38)		
Difference	0.48 (0.85)	-0.94	0.35
**PTSD (DTS)**			
Placebo	15.04 (1.70)		
Pregnenolone	17.71 (1.93)		
Difference	-2.67 (1.91)	1.20	0.23
**Executive Function (TOL)**			
Placebo	19.58 (0.22)		
Pregnenolone	19.04 (0.30)		
Difference	0.54 (0.72)	-1.45	0.15
**Physical Functioning (RAND-36)**			
Placebo	65.56 (1.45)		
Pregnenolone	62.30 (2.50)		
Difference	3.26 (1.99)	-1.27	0.21
Model estimates converted back to data scale. Abbreviations: AIS=Athens Insomnia Scale, CD-RISC=Connor Davidson Resilience Scale; DS=Digit Sequencing; DTS=Davidson Trauma Scale; TOL=Tower of London; RAND=Physical Functioning scale of the RAND-36.			

eTable 4. Neurosteroid Level Changes by Treatment Group

	N	Placebo	N	Pregnenolone	p-Value
Pregnenolone (pg/mL)					
Visit 3 (Baseline)	49	585.26 ± 48.85	45	585.26 ± 48.85	0.99

© 2020 Naylor JC et al. *JAMA Network Open.*

Visit 4	48	599.68 ± 54.70	45	1352.50 ± 107.57	0.000
Visit 5	47	684.04 ± 63.02	45	2443.89 ± 216.11	0.000
Visit 6	42	616.73 ± 51.20	41	2786.24 ± 285.94	0.000
Allopregnanolone (pg/mL)					
Visit 3 (Baseline)	49	62.22 ± 5.10	45	67.96 ± 9.83	0.95
Visit 4	48	59.73 ± 5.64	45	508.98 ± 55.31	0.000
Visit 5	47	71.22 ± 11.07	45	1036.50 ± 108.04	0.000
Visit 6	42	61.32 ± 6.00	41	1263.10 ± 136.90	0.000
Pregnanolone (pg/mL)					
Visit 3 (Baseline)	49	32.75 ± 2.02	45	34.98 ± 25.19	0.98
Visit 4	48	30.72 ± 2.58	45	249.13 ± 29.07	0.01
Visit 5	47	35.22 ± 3.68	45	679.00 ± 86.34	0.000
Visit 6	42	32.84 ± 2.28	41	883.87 ± 166.57	0.000
Androsterone (pg/mL)					
Visit 3 (Baseline)	49	177.60 ± 10.92	45	171.67 ± 14.06	0.70
Visit 4	48	171.89 ± 9.55	45	163.12 ± 12.73	0.58
Visit 5	47	177.42 ± 9.23	45	149.22 ± 11.0	0.08
Visit 6	42	186.26 ± 12.32	41	148.93 ± 11.07	0.02
p-Value is calculated from the difference between placebo- and pregnenolone-treated conditions.					

© 2020 Naylor JC et al. *JAMA Network Open.*
