# Supplementary material for: Effect of Pregnenolone vs Placebo on Self-reported Chronic Low Back Pain Among US Military Veterans: A Randomized Clinical Trial
Source: JAMA Netw Open. 2020 Mar 2;3(3):e200287. doi: 10.1001/jamanetworkopen.2020.0287 (PMC7052727; doi:10.1001/jamanetworkopen.2020.0287)
Supplement: Supplement 3. — Data Sharing Statement [file jamanetwopen-3-e200287-s003.pdf]

Naylor JC, Kilts JD, Shampine LJ, et al. Effect of pregnenolone vs placebo on self-reported chronic low back pain among US military veterans: a randomized clinical trial. JAMA Netw Open. 2020;3(3):e200287. doi:10.1001/jamanetworkopen.2020.0287

Data Sharing Statement

Data

Data available: No

Additional Information

Explanation for why data not available: Data will be released according to VHA guidelines, upon appropriate request
